# The school environment and adolescent physical activity and sedentary behaviour: a mixed‐studies systematic review

**DOI:** 10.1111/obr.12352

**Published:** 2015-12-18

**Authors:** K. L. Morton, A. J. Atkin, K. Corder, M. Suhrcke, E. M. F. van Sluijs

**Affiliations:** ^1^ UKCRC Centre for Diet and Activity Research (CEDAR), MRC Epidemiology UnitUniversity of CambridgeCambridgeUK; ^2^ Centre for Health EconomicsUniversity of YorkYorkUK

**Keywords:** Adolescent, physical activity, school, sedentary behaviour

## Abstract

There is increasing academic and policy interest in interventions aiming to promote young people's health by ensuring that the school environment supports healthy behaviours. The purpose of this review was to summarize the current evidence on school‐based policy, physical and social‐environmental influences on adolescent physical activity and sedentary behaviour. Electronic databases were searched to identify studies that (1) involved healthy adolescents (11–18 years old), (2) investigated school‐environmental influences and (3) reported a physical activity and/or sedentary behaviour outcome or theme. Findings were synthesized using a non‐quantitative synthesis and thematic analysis. Ninety‐three papers of mixed methodological quality were included. A range of school‐based policy (e.g. break time length), physical (e.g. facilities) and social‐environmental (e.g. teacher behaviours) factors were associated with adolescent physical activity, with limited research on sedentary behaviour. The mixed‐studies synthesis revealed the importance of specific activity settings (type and location) and intramural sport opportunities for all students. Important physical education‐related factors were a mastery‐oriented motivational climate and autonomy supportive teaching behaviours. Qualitative evidence highlighted the influence of the wider school climate and shed light on complexities of the associations observed in the quantitative literature. This review identifies future research needs and discusses potential intervention approaches to be considered.

## Background

Physical activity in young people is associated with improved cardiovascular health 1, mental health 2 and academic performance 3. Despite these established benefits, a substantial proportion of young people fail to meet physical activity guidelines. Moreover, participation declines during the transition from childhood to adolescence 4, 5, with physical activity increasingly replaced with sedentary activities 6. Given that young people spend approximately half of their waking day at school, schools represent an important setting for promoting physical activity and reducing sedentary behaviours.

Overall, school‐based physical activity interventions have tended to focus on increasing knowledge via health education and printed/audio‐visual materials and implementing curricula to increase the amount of time students are engaged in physical activity during the school day. Generally speaking, these interventions have not been successful for adolescent populations 7. A general criticism directed at many school‐based interventions is the lack of attention paid to the role of the wider school environment 8. A growing body of research suggests that human behaviour is not only driven by deliberation (e.g. knowledge, attitudes and beliefs) but can also be automatic, cued by environmental stimuli 9. These environmental factors may be physical (e.g. physical structures and facilities), social (e.g. social support and social norms) or institutional (e.g. within‐school rules and policies). This is consistent with ecological approaches to behaviour change, which posits that environments restrict the range of behaviour by promoting and sometimes demanding certain actions and by discouraging or prohibiting others 10.

Both in academia and policy, there is increasing interest in identifying interventions that aim to promote young people's health by ensuring that the wider school environment supports healthy behaviours 11. A recent Cochrane review 12 examined the evidence for the ‘health promoting schools’ (HPS) framework, which combines (a) the school's social and physical environment, (b) health education within the formal school curriculum and (c) links with families and the wider community. On the whole, an HPS approach demonstrated effectiveness for physical activity promotion. However, limited conclusions can be drawn regarding the specific role of the school environment as interventions combined environmental modifications with traditional health education and/or family involvement. Another review focussing exclusively on the school environment facet of HPS 11 found that environmental interventions show potential for increasing physical activity. This review included intervention studies only, with just two studies targeting physical activity in adolescent populations. Indeed, it has been highlighted that the lack of research into HPS approaches for adolescent populations represents a ‘missed opportunity for public health impact’ 13 (p. 15).

Given that the aforementioned reviews 11, 12 and others 14, 15, 16 examine evidence across childhood and adolescence (e.g. 5–18 years old), it is unclear how the associations observed operate among different age groups. For example, two recent reviews have examined the impact of playground designs 17 and physically active lessons 18. Both provide encouraging evidence in favour of these approaches, but only one secondary school‐based intervention was included across both reviews. It is therefore unknown whether these types of environmental interventions that appear to be effective in primary schools may also be beneficial for adolescent populations.

With such limited experimental evidence available, the inclusion of multiple forms of evidence is crucial to identify potentially effective approaches that have yet to be tested. As such, the objective of this mixed‐studies systematic review was to provide information on what school‐environment factors are associated with adolescent physical activity and sedentary behaviour.

## Methods

### Study identification

Four electronic databases were searched in June 2014 (PubMed, Web of Science, PsycINFO, ProQuest [including British Education Index; Australian Education Index; ERIC]). The search strategies are shown in the Supporting Information (Table S1). No date limits were applied. Reference lists of included studies and of relevant reviews (e.g. 11, 12, 15, 19, 20) were searched for further publications. The following inclusion criteria applied were (1) involving healthy adolescents (11–18 years old), (2) investigating the influence of the school environment and (3) reporting a physical activity and/or sedentary behaviour outcome measure or theme.

Following the searches, all results were exported into a reference manager and duplicates removed. Initially, titles and abstracts were screened by the first author (K. M.) for obvious irrelevance; 15% were double checked by another author (E. v. S). In the next phase, full text versions of selected articles were obtained, and inclusion and exclusion criteria assessed. At this stage, all articles were screened by at least two authors (K. M., A. A. and E. v. S.). Any disagreements were resolved in a meeting involving all three authors.

### Inclusion and exclusion criteria

Studies could be set in secondary schools and/or middle schools (mean age of participants >11 years old). We excluded primary school‐based studies and those examining clinical populations only (e.g. youth with physical or mental disabilities, or students with asthma or diabetes).

Consistent with an ecological approach, we defined environment as the physical and aesthetic surroundings of the school and/or the psychosocial climate and culture of the school. In this sense, environment refers to the wider ‘ethos’ of the school relating to physical activity, including physical activity‐specific policies (e.g. organisational statements or rules that are meant to influence behaviour), school organisation/management; teaching; discipline; pastoral care and features of the physical environment 11. Experimental studies that focussed predominantly upon changing individual‐level factors (e.g. health education, behavioural skills training and motivational interviewing) were excluded. We also excluded multi‐component interventions, which included a significant health education or family/community approach.

Quantitative studies were included if they reported on a physical activity or sedentary behaviour outcome measured by self‐report or proxy‐report questionnaire or objectively measured. Qualitative studies were excluded if they did not provide a theme that is related to how physical activity is influenced by the school environment.

### Data extraction (and selection)

Standardized forms were used to extract data from the selected studies. This included (a) author, year of publication and country, (b) aims of the study, (c) participant characteristics, (d) study characteristics/context, (e) intervention components/exposure measures (quantitative studies only), (f) primary outcomes (quantitative) or themes (qualitative) and (g) any cost/cost‐effectiveness data available. Relevant data were extracted by the first author (K. M.) and double‐checked by a second author (A. A. or K. C.). Discrepancies were resolved through a consensus discussion.

### Risk of bias (quality) assessment

Each included article was quality‐assessed using a modified tool appropriate for mixed‐studies reviews 21. This tool assesses quantitative observational studies using items that reflect the appropriateness of the sampling, the justification of the measures and the control of confounding variables. Quantitative experimental studies are assessed according to the appropriateness of randomisation, blinding and complete outcome data. Finally, qualitative studies are assessed according to the appropriateness of the qualitative approach, description of the context, the justification of the sampling and the descriptions of the data collection and analyses. This tool is shown in the Supporting Information (Table S2), including the items that were added for the purpose of this review and the scoring strategy adopted. Specifically, we added items to be able to distinguish between the quality of the observational design (e.g. cross‐sectional or prospective), the quality of the exposure variables (for the observational studies) and the nature of the physical activity outcome assessment (e.g. subjective or objective) used in the quantitative studies.

### Synthesis

Given that a large proportion of the included studies were focussed exclusively on the physical education (PE)‐specific environment, we split the findings into studies that addressed the ‘whole school’ environment (e.g. school grounds and extra‐curricular physical activity policies) and those that addressed the ‘PE’ environment only (e.g. size of PE instructional area and PE teacher behaviours). Environmental characteristics were grouped into broad categories, (1) physical environment, (2) social environment and (3) policy environment, to aid the presentation of results and facilitate evidence synthesis.

For the quantitative data, we performed a non‐quantitative narrative synthesis of all reported correlates of activity. Heterogeneity of methods used to assess physical activity and sedentary behaviour, along with contrasting definitions and measures of the school environment, precluded synthesis by meta‐analysis. Similar exposures were combined (e.g. ‘frequency’ and ‘hours’ of PE provision). Associations were extracted for the smallest available subgrouping (e.g. sex and age). Where multiple stratifications were presented, data for subgroups based on sex were prioritized. If studies were reported on multiple outcomes, data for the most comprehensive measure (e.g. total physical activity) were used. Data on a second outcome was only included where this is related to sedentary behaviour. For each potential correlate, associations from individual studies/samples were categorized as ‘−’, significantly associated with lower physical activity, ‘0’, no significant association/effect or ‘+’, significantly associated with higher physical activity. Reverse coding was used for those studies reporting on sedentary outcomes. Consistency across studies was then summarized using a previously applied algorithm 22 labelled as: ‘+’ or ‘−’ or ‘0’ if 60–100% of the studies reported the same direction, or ‘?’ (indeterminate/possible) if fewer than 60% of the studies (for each correlate) reported a consistent direction. Moreover, where four or more studies reported on a potential association, double signs were used to indicate greater confidence in the summary (e.g. ‘00’, ‘??’, ‘++’, and ‘− −’).

Qualitative data were synthesized and analysed thematically using NVivo in three stages, such as (1) line‐by‐line coding of primary studies; (2) organising codes into themes and (3) development of analytical themes 23. The final integrated synthesis consists of a narrative commentary for each facet of the school environment (physical, social and policy environment), which combines the results of quantitative and qualitative syntheses 24.

## Results

**Figure 1:**
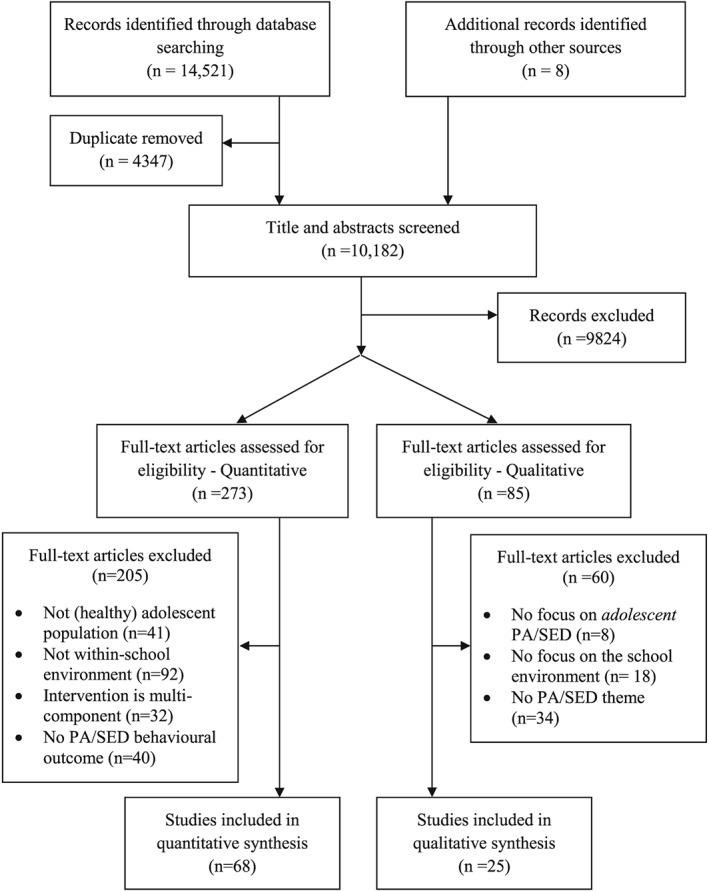
Flow diagram.

Ninety‐three papers (describing 91 different studies) met the inclusion criteria (see flowchart in Fig. 1). Sixty‐eight of these were quantitative studies and 25 were qualitative. Table 1 provides a brief overview of included studies. A more detailed description of each study is provided in the Supporting Information (Tables S3 and S4).

**Table 1:** Overview of characteristics of the studies included in systematic review of school

	Quantitative studies (*N* = 66)	N	Qualitative studies (*N* = 25)	*N*
Study design			N/A 38, 39, 40, 41, 45, 46, 47, 48, 49, 50, 51, 52, 53, 54, 55, 56, 57, 58, 59, 63, 64, 65, 66, 67, 78	25
Observational				
Cross‐sectional	31, 32, 33, 34, 35, 36, 37, 43, 60, 68, 69, 70, 71, 75, 76, 84, 85, 86, 87, 88, 89, 90, 91, 92, 93, 94, 95, 96, 97, 98, 99, 100, 101, 102, 103, 104, 105, 106, 107, 108, 109, 110, 111, 112, 113, 114	44		
Prospective	30, 31, 72, 73, 74, 90, 115, 116, 117, 118, 119	11		
Experimental				
RCT/cluster RCT	25, 26, 29, 61, 120	5		
Other experimental*	27, 28, 77, 121, 122, 123	6		
Study location				
North America	26, 30, 32, 33, 34, 36, 37, 43, 61, 68, 69, 71, 76, 84, 85, 86, 87, 90, 95, 101, 102, 104, 105, 107, 109, 110, 113, 114, 115, 117, 120, 123	31	38, 39, 47, 54, 55, 57, 59, 65, 66, 78	12
Europe (excluding UK)	28, 72, 73, 75, 77, 88, 89, 91, 92, 93, 94, 108, 116, 119	14	51	1
Australia and New Zealand	25, 31, 35, 60, 70, 98, 100, 103, 121, 122	10	53, 56, 58, 64	4
Asia	72, 73, 74, 96, 97, 106, 111, 112	7		
UK	27, 29, 72, 118	4	41, 45, 46, 48, 49, 52, 63, 66	8
Central America			50	1
Sample size†				
Quantitative				
<100 participants	88, 99, 122, 123	4		
100–299 participants	25, 27, 29, 30, 34, 69, 73, 76, 77, 86, 101, 103, 109, 114, 116, 118	16		
300–999 participants	28, 71, 72, 74, 75, 84, 87, 90, 91, 112, 113, 115, 121	14		
1000–9999 participants †	26, 31, 32, 33, 35, 36, 37, 60, 61, 68, 70, 92, 93, 94, 95, 96, 97, 98, 100, 105, 106, 107, 108, 111, 117, 120	27		
*≥*10,000	43, 85, 89, 102, 104, 110	6		
Qualitative				
<30 participants			39, 40, 45, 51, 52, 53, 63, 67	8
30–99			46, 47, 48, 54, 55, 56, 57, 58, 59, 64, 65, 66, 78	13
≥100			38, 41, 49, 50	4
Schools targeted				
Quantitative				
Middle school	26, 30, 32, 33, 34, 36, 37, 61, 68, 71, 76, 84, 85, 91, 95, 104, 105, 109, 112, 113, 114, 120, 123	23	38, 39, 54, 59, 67, 78	6
Secondary school (e.g. high school and junior high)	25, 27, 28, 29, 31, 32, 35, 43, 60, 70, 72, 73, 75, 77, 87, 89, 90, 92, 93, 94, 96, 97, 98, 100, 102, 103, 106, 108, 110, 111, 115, 116, 117, 118, 119, 121, 122	37	40, 41, 45, 46, 47, 48, 49, 50, 51, 52, 53, 55, 56, 57, 58, 63, 64, 65, 66	19
Combined schools	88, 93, 99	3		
Not specified	69, 86, 101, 107	4		
Environment addressed				
Whole school environment	26, 27, 31, 32, 33, 34, 35, 43, 60, 61, 68, 69, 70, 71, 84, 85, 86, 87, 88, 89, 90, 91, 92, 93, 94, 95, 96, 97, 98, 99, 100, 101, 102, 103, 104, 105, 106, 107, 115	39	38, 39, 45, 46, 47, 48, 49, 50, 53, 54, 55, 56, 57, 58, 59, 63, 64, 78	18
PE environment only	25, 28, 29, 30, 36, 37, 72, 73, 74, 75, 76, 77, 108, 109, 110, 111, 112, 113, 114, 116, 117, 118, 119, 120, 121, 122, 123	27	40, 41, 51, 52, 65, 66, 67	7
Physical activity outcome				
Within school PA/SED				
Total PA in school day	27, 31, 35, 43, 68, 69, 70, 73, 76, 84, 87, 89, 90, 96, 97, 101, 102, 106, 107, 108, 109, 114, 115	23		
Within‐class (PE) PA/SED	25, 30, 36, 37, 77, 104, 109, 110, 111, 112, 113, 117, 120, 121, 122, 123	16		
After‐school/extracurricular PA	26, 32, 60, 95, 98, 102	6		
PA during lunch/breaks	60, 88, 92, 93, 94, 103	6		
Leisure time PA/SED	28, 29, 71, 72, 73, 74, 75, 91, 98, 100, 105, 116, 117, 118	14		
Active transport	60	1		
Physical activity outcome (intensity)				
Overall PA‡	27, 28, 31, 34, 35, 36, 37, 60, 68, 71, 74, 75, 88, 91, 95, 96, 97, 98, 99, 100, 101, 102, 106, 108, 109, 111, 112, 113, 115, 117	30		
MVPA	25, 26, 30, 32, 33, 35, 43, 61, 69, 70, 73, 76, 77, 84, 85, 87, 89, 90, 92, 93, 94, 103, 104, 105, 107, 109, 114, 115, 120, 121, 122, 123	31		
Vigorous PA	26, 29, 72, 73, 92, 93, 94, 102, 110, 115, 116, 118, 119, 121	14		
Light PA	33, 103, 110	3		
SED	25, 86, 103	3		
Physical activity outcome (measure)				
Subjective (e.g. questionnaire/proxy ratings)	26, 27, 28, 29, 31, 43, 60, 68, 69, 70, 71, 72, 73, 74, 75, 76, 84, 85, 86, 87, 89, 90, 91, 92, 93, 94, 95, 96, 97, 98, 100, 101, 102, 106, 107, 108, 109, 110, 114, 115, 116, 117, 118, 119	44		
Objective (e.g. pedometer/accelerometer/heart rate monitoring and observation)	25, 30, 31, 32, 33, 34, 35, 36, 37, 61, 69, 77, 88, 91, 99, 103, 104, 105, 109, 112, 113, 120, 121, 122, 123	25		

* This includes one quasi‐experimental design 27 and one single‐subject reversal design 123.

† This includes studies in which the classes or schools were the target (not individual children), therefore accurate numbers of individual children are not provided 36, 37, 61, 104, 111, 120.

‡ Overall PA includes ‘involvement in structured activities’ 68 and ‘school sports participation’ 95. This also included studies that combined intensities into one PA value or did not specify the intensity examined.

MVPA, moderate to vigorous physical activity; RCT, randomized controlled trial; PA, physical activity; PE, physical education.

Overall, most studies were published within the last 5 years (68%) and conducted in North America (primarily the USA). Almost two‐thirds of the included studies (62%) targeted the whole school environment, with approximately 38% exclusively targeting the PE environment. The majority of quantitative studies had sample sizes of >1000 participants, with several studies including >10,000 participants. The majority of qualitative studies included between 30 and 100 participants. Regarding the quantitative studies, most were cross‐sectional (68%), 11 used a prospective designs (16%) and 11 were experimental (16%) including five randomized controlled trials (RCTs). Physical activity was assessed using subjective methods in 43 (63%) studies, whilst objective assessment was featured in 25 studies (37%). Most assessed either total physical activity or moderate to vigorous physical activity (>90%) with very few studies addressing light physical activity (*n* = 3) or sedentary behaviour (*n* = 3).

### Quality assessment

#### Quantitative studies (see Table S3 in the Supporting Information)

Most of the 11 experimental studies were of moderate quality; only one PE‐based study was deemed of high quality 25. This study randomized students to different teaching strategy interventions, blinded students to alternative intervention options and also assessed intervention fidelity using blinded assessors. This study also utilized an objective physical activity measure, which was where a number of other experimental studies fell short 26, 27, 28, 29. Studies deemed of low quality tended to be small pilot designs, which did not adopt randomisation (e.g. 27) or where complete outcome data was not reported (e.g. 28). Overall, the observational studies were of mixed quality. Few adopted prospective designs (*n* = 11), of which only two utilized objective measures of physical activity 30, 31. Higher quality cross‐sectional studies tended to adopt objective activity measures, objective/valid exposure measures and adequately control for confounding (and adjust for nested data if relevant).

When stratifying the studies by type of environment, it appeared that those focussed on the physical environment of schools tended to be mostly cross‐sectional. However, in this group, those rated as high quality all adopted objective measures of physical activity 32, 33, 34, 35, 36 and utilized objective exposure measures where possible 33, 34 (e.g. geographic information system (GIS) techniques to map campus characteristics). Few studies appropriately accounted for potential confounding. In contrast, the group of studies focussed on the social environment included relatively more prospective and experimental studies (mostly relating to PE teaching behaviours), but the majority used self‐report outcome measures. Lastly, studies focusing on the school's policy environment tended to adopt larger sample sizes across multiple sites; however, the majority of these also adopted self‐report outcome measures. Only two included objective measures of physical activity in relatively large sample sizes 32, 37.

#### Qualitative studies (see Table S4 in the Supporting Information)

Most included qualitative studies were of moderate quality, with four of high quality 38, 39, 40, 41. High‐quality studies tended to include an appropriate and specific qualitative approach for the topic in question (and a sound justification of this approach) as opposed to the more generic ‘qualitative approach’ reported in most studies. Furthermore, these tended to describe the participants in greater detail and include a justification for the sampling and recruitment method used (e.g. purposive sampling). In addition, they provided a richer description of study context, data collection and analyses, which establishes credibility of the findings 42. Very few studies included details on researcher reflexivity (e.g. an awareness of the researcher's own biases and role within the study) or included methods to establish trustworthiness of the data, such as triangulation or the use of multiple analysts.

### Physical environment

#### Quantitative studies

Nineteen papers (describing 17 unique studies) included exposure measures that correspond to the whole school's physical environment (Table 2). A total of eight unique physical environmental exposure variables were investigated, half of which were only investigated in one or two studies. The only physical environmental factor consistently positively associated with physical activity was the ‘activity setting’ (the type and location for specific activities, e.g. baseball field, indoor gym etc.). For example, in one study, having an ‘alternate room for physical activity’ was associated with greater activity, even though the total number of facilities was not 43, 44. ‘Access to physical activity or sports equipment’ was consistently not associated with physical activity, although in two studies, positive associations were shown for boys only. ‘Physical activity facilities’ and ‘physical activity area/field size’ showed indeterminate associations. Of the less frequently studied factors, only the campus area size per student showed a potential positive association with physical activity. All other exposures showed an indeterminate or no association.

**Table 2:** Whole school environment (observational and experimental studies)

	Association with PA/SED			No. of samples	Summary
Factor	**−**	0	**+**		
Physical					
Activity setting (type, location)		32g, 104b	32b, 88, 104g	5	++
PA facilities (access, number)	98	43b*, 43g*, 87, 91b, 91g, 97b, 97g, 104b	33, 35, 85, 92, 93, 101, 104g	16	??
Field/PA area size		33, 104b	34,104g	4	??
Campus area per student			34	1	+
School building area per student		33	34	2	?
Access to sports/PA equipment		61g, 86, 86^TV^, 104g	61b, 104b,	6	00
School design (greenery)		99		1	0
Overall school PA friendliness		71		1	0
Social					
Perceived school PA climate/support					
‐ Overall		68, 69, 70, 71	26, 60j, 60s	7	??
‐ Teacher		90	84, 90, 100	4	++
‐ Boys		84		1	0
Adult supervision		32b, 32g, 61g, 91b, 104b, 104 g,	91g, 61b	8	00
School social capital (e.g. connectedness)		100	85, 107	2	+
Policy					
Number of PA policies			89	1	+
Extracurricular PA activities		61g, 86, 86^TV^,91g, 96, 97 g, 104b, 104g,	26, 61b, 91b, 94, 97b, 98,, 106b, 106g	14	??
Intramural vs. interscholastic sports		32g	32b	2	?
‐ School offers intramural sport		43b	43g, 95, 115	4	++
‐ School offers interscholastic sport		43b, 43g, 115		3	0
PE provision (d/frequency/h)	97g	31b, 31g, 43b, 43g, 94, 97b, 102, 106b	86, 106g	11	00
Active lessons		27		1	0
Access to field/play area out‐of‐school		86^TV^, 105	86^PA^	3	0
School involved in PA promotion project		94		1	0
Allowing students to cycle to school	97b	97g		2	?
Quality of sports management			98	1	+
Break time length		103		1	0
Recess exercises			106b, 106g	2	+

* This citation 43 includes additional papers 44, 124 as these report on the same overall study.

E, experimental studies; P, prospective studies; C, cross sectional studies – cross‐reference in Supporting Information Tables S3 and S4; PA, physical activity; all variables are PA unless stated; SED, sedentary time/behaviour; TV, TV watching; b, boys; g, girls; j, junior; s, senior. Direction of association: −, significantly lower PA/higher SED; 0, no significant difference; +, significantly higher PA/lower SED. For **≤**3 studies: ‘?’ if 34–59% support a specific association, and ‘+’, ‘−’ or ‘0’ if 60–100% support a positive, negative or no association. For **≥**4 studies: ‘++’, ‘—’ or ‘00’ if 60–100% support positive, negative or no association and ‘??’ if <60% of studies support a specific association.

Only two observational studies investigated physical environmental factors related to the PE‐specific environment (Table 3), investigating three unique exposure variables. Only ‘size of instructional area’ showed a potential positive association.

**Table 3:** PE environment (observational and experimental studies)

Factor	Association with PA/SED			No. of samples	Summary
	−	0	+		
Physical					
PE lesson location		111		1	0
Size of instructional area			111	1	+
Indoor vs. outdoor lesson		36	36^SED^	2	?
Social					
‘PA promotion teaching behaviours’ *		61g, 112	61b, 120	2	?
‘Teacher influence’†	108			1	−
Social support			114	1	+
Positive feedback			119	1	+
Provision of choice			25, 25^SED^ 121	2	+
Active supervision			123	1	+
Transformational teaching behaviours			117	1	+
Psychological need support (autonomy, competence and relatedness support)			76	1	+
‐ Autonomy support			29, 72, 74, 75, 116, 118, 122	7	++
‐ Relatedness support			74	1	+
Motivational climate					
‐ Perceptions of learning/mastery climate		109	28, 30, 73, 113, 116	5	++
‐ Perceptions of performance climate		30, 109, 116		3	0
Policy					
State policies					
‐ PE requirement binding		110b, 110g		2	0
‐ PE goals set		110g	110b	2	?
‐ Schools must offer PE		110b, 110g		2	0
‐ School give PE test		102, 110b, 110g		3	0
‐ PE exemption for sport		110g	110b	2	?
‐ PE exemption for other reason		110b, 110g		2	0
Single sex vs. co‐ed		37b, 37g, 77		3	0
PE class size	36, 36^SED^	111		3	−

* This includes teaching behaviours assessed by the SOFIT observational tool (feedback, prompts/cueing/demonstration/out of class PA promotion/no PA promotion).

† This includes role‐modelling, social support and social influence.

E, experimental studies; P, prospective studies; C, cross sectional studies – cross reference in Supporting Information Tables S3 and S4; PA, physical activity; all variables are PA unless stated; SED, sedentary time/behaviour; b, boys; g, girls. Direction of association: −, significantly lower PA/higher SED; 0, no significant difference; +, significantly higher PA/lower SED. For **≤**3 studies: ‘?’ if 34–59% support a specific association, and ‘+’, ‘−’ or ‘0’ if 60–100% support a positive, negative or no association. For **≥**4 studies: ‘++’, ‘—’ or ‘00’ if 60–100% support positive, negative or no association and ‘??’ if <60% of studies support a specific association.

#### Qualitative studies

Features of the school's physical environment were discussed in 16 qualitative studies. Factors that were highlighted as potential influences on physical activity and sedentary behaviour included the school's facilities, space and equipment. These were predominantly discussed as the lack of facilities and/or the poor quality of school's physical activity facilities 38, 45, 46, 47, 48, 49, 50, 51, 52.

…some schools have soccer fields and basketball courts, but there's none of that here. During recess, we play soccer in the hallway, but the principal won't let us play there . . . 50

Poor changing facilities 47, 49 and the lack of bike storage facilities 48 were also cited as negative influences on activity. Similarly, the lack of a ‘playground’ was mentioned as limiting intrinsic motivation to ‘play’^51^, although another study highlighted that students reported being ‘too old’ for playgrounds and held the perception that ‘safe’ play spaces were ‘boring’ 53. Six studies referred to ‘space’ as an important factor. Spacious environments were suggested to promote physical activity 53, with several studies identifying a lack of ‘space’ at school as limiting activity 54, 55 or suggesting that existing space could be improved to promote greater activity 56. Only one study included a reference to the school's building design, in that it positively impacted upon activity because of the number of stairs 49. Finally, a lack of, or out‐dated/poor equipment was frequently highlighted as a negative influence on physical activity 38, 53, 55, 56, 57, 58, 59.

Like in our primary school they had a sport shed and you could borrow something, write your name and borrow something, they should have that here, but they don't 56

A limited level of detail provided within the included papers prevents developing a deeper understanding of why the school's physical environment impacts upon adolescent physical activity and sedentary behaviour. There was a suggestion that the lack of physical infrastructure within the school meant that more physical activity opportunities could simply not be offered (i.e. the physical environment limits the policies that can be established) 46. Moreover, there were suggestions that the lack of equipment available meant that students had nothing to do at break times 60, that poor facilities undermine intrinsic motivation in PE 52 and that the school's existing space possesses a greater number of ‘sedentary rather than physical activity opportunities’ ([53 p. 12]).

…we always hang out in the student lounge…because there is a plasma TV and you watch all the music videos and stuff 53

#### Summary (mixed study synthesis)

Taken together, this review provides support for the importance of specific settings for physical activity within schools. That is, it may not be the number of or access to facilities in general that is of importance, but rather the availability of specific facilities that are perceived as adequate and accessible by students. The importance of sufficient space (in the PE‐specific environment and at the whole school level) was also considered important for physical activity, as highlighted in the quantitative and qualitative studies. A lack of equipment (or poor quality equipment) was discussed in several qualitative studies as a prominent barrier to physical activity at school; however, the findings from the quantitative studies indicate that this may be most relevant for boys. The qualitative evidence highlighted the dominance of physical features that might encourage sedentary behaviour, but this has yet to be tested quantitatively. Furthermore, the qualitative studies indicate that the influence of the physical environment on physical activity is a complex process, which also closely ties to the school's policy environment (i.e. whether the school allows access to the facilities). If a school with excellent equipment and multiple facilities does not provide (extra‐curricular) opportunities for these to be utilized, the impact of the physical features on adolescent physical activity is likely to be compromised.

### Social environment

#### Quantitative studies

Thirteen observational studies and two experimental studies investigated a total of five unique social environmental factors pertaining to the whole school environment (Table 2); three were studied four or more times. The ‘perceived overall school physical activity climate/support’ showed an indeterminate association, whereas a whole school's ‘social capital’ (e.g. how connected individuals feel to their school) showed a potentially positive association. Interestingly, ‘perceived teacher support’ showed a consistent positive association, but ‘adult supervision’ consistently showed no association. Two experimental studies included social environmental intervention components as part of wider environmental strategies. The first focussed on changing PE teaching behaviours and increasing levels of supervision 61, whereas the second focused on changing the school's social environment through provision of training to empower members of the school community (adults and youth) to create school environments that promote physical activity 26. Here, a positive social environment was characterized by fostering ‘connection’, ‘autonomy’, ‘skill building’ and ‘healthy norms’. Both showed positive effects on physical activity, albeit only in boys in the first instance.

Within the PE environment, fourteen observational and eight experimental studies investigated a total of 11 social environmental factors (Table 3), all broadly related to the PE teacher's behaviour. Two of these factors were studied four or more times. ‘Motivational climate’ refers to the climate created within the lesson (by the teacher), which can either be ‘mastery’ focussed (e.g. students perceive they are rewarded for learning and improvement) or ‘performance’ focussed (e.g. students perceive they are rewarded for superior performance over others) 62. A mastery climate was consistently associated with greater activity, both within and outside of PE. Related to this, teacher ‘autonomy support’ (e.g. teacher provides support for self‐initiation, choice, independent problem‐solving and involves the student in decision making) and ‘provision of choice’ within PE also showed consistent associations with physical activity and sedentary time. Other aspects of PE teacher behaviours were assessed in single studies; most showed positive associations with physical activity.

#### Qualitative studies

Eighteen qualitative studies discussed features of a school's social environment in relation to physical activity and sedentary behaviour. Factors that were highlighted as important included the school's ethos and culture surrounding physical activity, the school and PE ‘climate’ and specific PE teaching behaviours.

The most prominent theme to emerge was the negative influence of a competitive ethos or competition‐focussed‐climate 39, 45, 54, 56, 57, 58, 63, 64 or simply the ‘seriousness of participation’ 58. It was suggested that this can reduce participation, especially for girls and/or students of lower athletic ability 54. In one study, teachers reported that there was a desire to encourage elite performance and raise the profile of the school through sporting excellence.

As a teacher in lessons I think you want maximum [participation], but outside of lessons and the development of talent, I think you want to go elitist. So it's a bit of, that old dilemma. 45

The wider culture of the school surrounding physical activity was discussed in several studies. In one study, students, teachers, principals and parents recognized that how a school approached the notion of physical activity had an important impact on how active the people in the school were. As one teacher reported:

…the whole school atmosphere, I think it really does promote and want kids to be involved [with physical activity].... 39

The wider school culture was also discussed in terms of the level of priority given to physical activity. In one study, heads of PE and heads of school year discussed how their schools undervalued physical activity, as demonstrated through a lack of volunteering by other staff to support extracurricular opportunities and teachers being ‘unwilling to give up their time at either lunch time or after‐school’ 45. Another study discussed how PE is given a much lower priority than other ‘academic subjects’. This (coupled with poor funding and scarce resources) forces programmes to compete against each other, which ultimately degrades the school culture surrounding physical activity 39.

Students in one study reported that being active at break and lunch time was not part of their school culture. This differed from primary school in which a culture of ‘play’ was supported.

Cause no‐one really does it like….all the boys… they used to play football but now none of them do so no one really runs about or anything. More people just sit there and talk. 63

Another prominent theme is related to teacher behaviours 39, 46, 48, 50, 55, 56, 57, 64. Findings consistently supported the importance of teachers providing encouragement 39, 46, 59 and support for physical activity 55, 59. Role modelling 39, 48, 50, 55, 56 was also highlighted as an important teacher behaviour. For example, one study, focused on active travel, reported that students felt that their teachers did not put into practice what may have been emphasized in lessons:

Basically all the teachers use their cars . . . our teacher when she goes to [local shop], that's right over there, during school time she always takes the car 48.

Some qualitative studies focussed exclusively on the PE environment. PE teacher's behaviours were discussed in several studies 51, 52, 65, 66, and the findings consistently highlighted the importance of several behaviours for facilitating motivation and participation within PE and involvement in physical activity in general. These included role modelling PA (i.e. actively taking part in lessons), encouragement and enthusiasm, caring and supportive behaviours and positive feedback 65, 66:

Miss G did influence my positive attitude towards PE because she was my first PE teacher in high school and really gave me her support [following an injury]… 65.

Teaching behaviours closely tie to the perception of the PE class climate, which also was discussed. Specifically, students in some studies reported a climate that had little emphasis on learning and improvement, in which attention is given to the most competent students and praise was only provided when students outplayed their peers 52. In different studies, some students (especially males) reported that the ‘competition’ element in PE was a positive and motivating factor for participation. Although females commented on positives of competition, they also discussed negative aspects, such as removing the ‘fun’ from activity 66, especially if the boys became “overly competitive” 67. Students reported that they would like teachers to make them feel more involved and give students more choice 40, 52; one study reported how girls restricted their engagement with PE when they perceived it as being gendered, unwelcoming to their participation and thus, not a choice 40. In a different study, female students reported that PE grading systems that focus on effort rather than skill increases effort and participation in PE 67.

Several hypotheses were raised that allow us to develop a deeper understanding of why the school's social environment impacts on physical activity and sedentary behaviour. As reported by MacQuarrie and colleagues 39 ‘placing higher value on athletic elitism can fracture the student population into subgroups, whose sense of belonging will vary depending on how much they feel they are important and connected to the school. Student judgements of belonging are core to motivating students' involvement in physical activity’ (p. 269). In separate studies, students also reported being put off by the focus on competition over participation and enjoyment 56, and that the use of exercise as punishment (often a feature of a ‘performance‐focussed’ climate) induces feelings of anger and injustice, lowering motivation rather than increasing effort 52. In contrast, a PE teacher's transformational leadership was believed to positively influence PE‐related beliefs, motivation and attitudes (e.g. enjoyment), PE teacher satisfaction and motivation to engage in out of school activities 65.

#### Summary (mixed‐studies synthesis)

The majority of the quantitative studies that focus on the school's social environment are confined to PE settings. However, the qualitative research indicates that the whole school environment is crucial, beyond what happens in PE lessons. Interestingly, the cross‐sectional studies that examined the school's overall support and/or climate for physical activity demonstrated an indeterminate association with physical activity. It is important to note that there was considerable variability in the exposure measures for school support and climate for physical activity. For example, measures to assess the school physical activity environment included measures of (girls') perceptions of teachers and boys influence on physical activity within the school 68, 69, the school's encouragement for PA 70 and overall teacher values surrounding physical activity 71. The inconsistent definition and measures of the school's social environment surrounding physical activity promotion makes it difficult to compare consistently across studies. Interestingly, one experimental study that focused on changing the wider social environment (relating to physical activity) demonstrated effectiveness 26. The focus of the intervention was on empowering students and adults at the school to create active opportunities. As reported in a qualitative paper, ‘the extent to which a school involves everyone from students through to administration in the planning and carrying out of ways to engage everyone in active lifestyles, the stronger the school culture on the promotion of physical activity’ 39 (p. 268). Taken together, the findings highlight the importance of wider school support and involvement in establishing a school culture that promotes physical activity for all, not just the competent and active students.

Within the PE‐specific environment, consistent support across quantitative and qualitative studies was shown for the importance of the class climate (e.g. a mastery/learning focused environment rather than a competitive environment) and PE teacher behaviours (e.g. autonomy supportive behaviours such as allowing students to make decisions, role modelling, support and encouragement for physical activity). A variety of mechanisms have been explored within the studies of the PE environment. For example, the impact of the PE environment (and PE teacher behaviour) on physical activity is mediated by several psychosocial variables, including (but not limited to) autonomous motives/intrinsic motivation 72, 73, 74, 75, 76 and a range of ‘efficacy’ beliefs such as increased self‐efficacy 26, 30 and increased proxy efficacy (i.e. the extent to which students feel confident to get others to act on their interests to create supportive environments) 26. These findings closely reflect the qualitative evidence relating to how and why the PE teacher behaviours impacted (either positively or negatively) upon their involvement in PE.

### Policy environment

#### Quantitative studies

Sixteen observational and three experimental studies investigated factors related to the whole school's policy environment. Of the 13 factors investigated (Table 2), only three were studied four or more times. ‘PE provision’ was consistently not associated with physical activity, whereas offering ‘extracurricular activities’ showed an indeterminate association. However, there was a suggestion that the latter may be more important for boys than girls, with three out of the four studies conducting stratified analyses showing a positive association in boys only. Studies investigating schools' policies about ‘intramural versus interscholastic sports participation’ showed that intramural sport was consistently associated with physical activity, whereas interscholastic sport showed no association. Of the factors studied less frequently, only ‘number of physical activity policies’, ‘quality of sports management’ and ‘recess exercises’ were potentially positively associated with activity, all other factors showed no or an indeterminate association.

All three ‘whole school’ experimental studies included policy components. Two focused on the implementation of organized activities/after‐school programmes as part of a wider intervention; both demonstrated positive effects on physical activity outcomes 26, 61. The third examined the impact of provision of active lesson content (implementing typical classroom tasks during brisk walking) 27. It showed no effect on self‐reported activity, although improvements in cholesterol and glucose levels were observed.

Four observational studies and one experimental study investigated factors that were related to the PE lesson's policy environment (Table 3), reporting on 10 distinct factors. Of these, eight were studied in one single study, all showing no or indeterminate associations across boys and girls. ‘PE class size' showed a potentially negative association indicating that students were less active and more sedentary in larger PE classes, whereas offering ‘single sex as opposed to co‐educational PE’ appeared not to be associated with physical activity. This latter finding was confirmed by an experimental study 77, which showed that both boys and girls were similarly active during single‐sex and combined games‐play lessons.

#### Qualitative studies

Features of the school's policy environment were discussed in almost all 22 of the 25 included qualitative studies. The majority focused on the provision of opportunities to be active during the school day. Studies highlighted the lack of opportunities for physical activity 59, poor range of sporting options for girls 64 or a lack of extracurricular sport for all 54, 58 as barriers to physical activity. This was specifically attributed to a lack of physical infrastructure and equipment in one study 54. Students also reported the need for more non‐competitive activities 38, 47, 54, 56 and emphasized participation rather than competition and exclusion 56.

Make it easier to get on the teams. You don't . . . really want to have competitiveness or otherwise half the kids won't do it. But you want to address certain kids, let them know that it's great, it's healthy for you. It's fun to get on a team 54

Some of the studies discussed the policies that limit physical activity during breaks. These included policies on which grades get to participate in outdoor recess 78, rules about accessing equipment during break times 38, 53, 56 and regulations regarding access to physical activity facilities during breaks and out‐of‐hours/after‐school (usually because of no supervision) 49, 53, 55. In one study, middle school teachers reported that open gym policies had positive effects on participation, although this appeared to benefit boys the most 54. Interestingly, adolescents in a separate study highlighted that too much supervision negatively impacted physical activity 53.

If there were too many teachers around, you wouldn't be able to do anything, so it would be boring 53

Active travel‐related policies were discussed in two studies 48, 54. One study highlighted that unsupervised active transport was generally discouraged because of a perception of ‘stranger danger’ and by the school's not providing crossing‐guards (‘lollipop man/lady’) 54. In another study, participants emphasized the need for cycle proficiency training and reward/incentive policies for students to promote active travel 48. The notion of schools implementing a rewards system for students being active was also mentioned elsewhere 59. Other wider school policies that created barriers to physical activity included physical activity uniforms (particularly for girls and related to body image concerns) 64, amounts of homework 78 and school scheduling that leaves students too tired or busy for physical activity 55, 56. However, in a different study, school scheduling was described (by a school principal) as being key to ‘crafting a culture of physical fitness’ 39 (p. 267):

It was built right into the program when the school was designed and the timetable was organized to facilitate intramurals. 39

Scheduling was also discussed in relation to PE, with one study highlighting that timetabling (avoid repetition of scheduling) PE class at the end of the day would facilitate participation in girls 47, because of their reluctance to change earlier in the day and worries about appearance. Students (and PE teachers) additionally perceived that they simply do not acquire enough PE 45, 47, 49, 52, 57, 78. Issues discussed included only requiring PE in certain grades in certain semesters 78, ‘active’ PE time taken up by taking notes and learning and large class sizes influencing PE quality (reported by parents) 38. Policies that allow exemptions for PE may be an additional barrier to learning and participation in PE 41, 56. Students suggested there should be more consequences for avoiding PE as they believed some pupils took advantage of this situation 56. Students, parents and schools believed that excuse notes provided a legitimate means to disengage from PE 40. This reinforces the perception that PE does not hold much value or priority. One teacher reported:

…often the parents' experience of PE is take a note and you are excluded. The hard thing is that as well as changing the pupils' perception of PE…for PE in schools to change, you know the parents need to be brought up to date as well… and again you're fighting the ‘PE isn't important’ status in schools 41.

The notion of separating classes by gender 39, 40, 47, 67 or ability 39 showed mixed opinions. Parents 47 and PE teachers 39 noted that separating class by gender would facilitate participation for girls. In contrast, adolescent girls were positive about co‐educational PE and felt that the interaction with boys fostered participation (effort) and skill development 40, 67. However, they reported negative feelings about co‐participation when boys created physically or emotionally unsafe learning environments 67. In a study of students identified as ‘motivated’ for PE, a desire for groups of similar ability was expressed 52.

In terms of developing a deeper understanding of why the school's policy environment impacts on physical activity, some papers provided additional insights. One argued that intramural activities helped to create an atmosphere of playfulness, encouraged those who do not want to take part in organized sports and signified the value of physical activity in the school 39. Generally speaking, school policies have the potential to create a school environment signalling that physical activity is important and a priority, or (as in most cases) that physical activity is not important and undervalued.

#### Summary (mixed‐studies synthesis)

Features of the policy environment were the most frequently discussed feature of the school environment within the qualitative studies. School policies appear to influence physical activity indirectly, mostly via the school's social environment to create a wider ‘culture’ of physical activity within the school. Several aspects of the school's policy environment highlighted in the qualitative studies were largely unexplored in the quantitative studies. These include the school's active travel policies (including incentives/rewards policies), uniforms and break time specific physical activity rules and regulations. The impact of only providing intramural sports opportunities has not been explored experimentally, and the support identified in both the observational quantitative and qualitative evidence suggests this may be a worthwhile avenue. Interestingly, whereas the (limited) amount of PE was highlighted as a barrier to activity in qualitative studies, the observational evidence consistently showed no association. The overall evidence also highlighted that whether co‐educational or single‐sex PE is beneficial is highly dependent on the wider learning environment (i.e. the social environment); specifically whether girls feel supported in their participation and comfortable with their skill level. Qualitative findings highlighted the interaction between the policy (i.e. co‐educational PE) and the social environment created in the PE class (i.e. motivational climate) and also emphasized the quality not quantity of PE. Taken together, the findings indicate a lack of independence and empowerment of the students, which is both encouraged by the school (e.g. through restrictive rules and regulations) and ultimately perceived by the students, thus negatively impacting upon their physical activity within school.

## Final synthesis and discussion

By applying a mixed‐studies approach and an inclusive definition of the school environment, we have been able to provide a comprehensive overview of the evidence on school‐environmental characteristics and adolescent physical activity and sedentary behaviour. Across the multiple forms of evidence, consistent support was found for (a) the importance of activity settings within school for physical activity, (b) the creation of a ‘culture’ of physical activity within the school, (c) teaching behaviours that support a positive climate for physical activity promotion, both within PE and beyond (e.g., role modelling, enthusiasm for physical activity and social support for physical activity) and (d) availability of intramural opportunities for all students.

In addition to those consistently supported, a multitude of other environmental factors have been explored. Several school‐environmental factors were highlighted in the qualitative evidence, yet remain untested quantitatively, such as school policies relating to school uniforms, break time (recess) rules and regulations (e.g. compulsory outdoor time or unsupervised access to equipment and facilities) and school active travel policies (e.g. incentives/rewards policies*)*. The qualitative evidence also emphasized the importance of overall school connectedness/cohesion (i.e. how connected student's feel to the school and their sense of belonging) and how the overall ethos and culture of the school influences student physical activity (demonstrated via supportive polices such as intramural opportunities and an ethos of inclusion rather than ‘elitism’). The influence of the wider school climate was explored in one RCT 26 with positive effects on physical activity. In light of the importance of the school's wider social environment identified in the qualitative evidence, there is a clear need for further experimental evidence examining the effect of modifying the school's wider social environment through the implementation of physical activity‐supportive policies. It also indicates a wider barrier to change within the school environment, in that features of the school's environment that are relevant to physical activity (and health more broadly) are seen as only applicable to PE settings. This finding is echoed in a recent review of Health Promoting School's initiatives; the emphasis on academic subjects (and the corresponding low value placed on health initiatives) and lack of wider institutional support are cited as major barriers to implementing physical activity initiatives that target the wider school environment 13.

The inclusion of qualitative evidence helped shed light on relatively mixed quantitative findings, specifically through highlighting the complexities that exist when considering how the school environment impacts upon physical activity. That is, it appears to be the combination and interaction of school‐environment factors that influence adolescent physical activity, rather than a single characteristic of the school. For example, although adequate facilities and equipment are considered important for physical activity promotion, if the wider school policies do not encourage and support the use of these by all students, the overall impact on physical activity is likely to be negligible. Furthermore, the arguments for and against co‐educational versus single‐sex PE policies highlighted in the qualitative studies appear to relate more to the motivational climate within the lesson and how supported students feel (e.g. through various teaching behaviours) rather than simply whether a lesson is single sex or co‐educational.

Across the quantitative and qualitative research, very limited attention has been given to how the school environment may promote or inhibit engagement in sedentary behaviours. In view of emerging evidence that sedentary behaviour may have independent health effects in young people 79, this is a topic worthy of further study. School‐environment interventions that seek to shift the distribution of activity intensity over the day may be more effective than a single focus on physical activity of a prescribed intensity 80. This argument, as well as increasing the frequency of interruptions to sedentary time, has been presented in other papers 81. This may require the development of novel intervention strategies (for adolescent populations) such as implementing activity breaks in class, the delivery of active lessons and changes to the classroom environment – all of which are under‐represented in the present review.

In recent years, studies in primary schools have successfully implemented changes to the school layout or classroom design, in order to ‘nudge’ pupils to walk more or substitute sitting with standing, regardless of demographic characteristics and motivation. For example, standing desks show some promising evidence for increasing calorie expenditure 82. Furthermore, building a new type of ‘activity permissive’ school environment designed specifically to encourage an active learning environment also showed promising results 83. Although innovative approaches to increasing physical activity and reducing sedentary behaviour in the school environment are being developed and tested, the evidence is largely confined to primary school settings. Whether these types of strategies are feasible, acceptable or effective for adolescent populations is unknown and worth exploring in future studies.

Lastly, feasibility and sustainability of intervention strategies are heavily influenced by their cost and cost‐effectiveness. Our search did not yield any results relating to the cost or cost‐effectiveness of the interventions. This limits the learning that can be applied in terms of appropriate directions for future studies and is an important area of future research. Furthermore, aside from a very small number of studies that examined differential effects based on gender, there was no examination of socio‐economic inequalities and the role of the environment (or environmental interventions) across different socio‐economic groups. It is important that school‐environment interventions to increase physical activity have the potential to reach individuals irrespective of individual characteristics or social circumstances.

A limitation of this review is that by only including studies that had a physical activity and sedentary behaviour outcome, we potentially missed a number of interventions that could potentially increase physical activity, but only included education or health‐related measures within their study. Although this review focused on physical activity and sedentary behaviour as the primary outcomes, it is important to note that none of the quantitative studies included any education‐focussed outcomes, such as concentration, cognitive functioning or behaviour in the classroom in addition to measures of physical activity. Furthermore, only one experimental study included wider health‐related outcomes (beyond anthropometry) 27.

### Future directions for creating active school environments for adolescent

With the exception of a very small number of experimental designs predominantly focussed on PE, there have been few attempts to modify adolescent's school environment. Future directions should include the development and testing of approaches identified in this review, through implementing a range of policies and addressing a wider cultural shift in relation to the priority given to physical activity within the school.

Future studies should also adopt rigorous outcome measures (objective where possible), include long term follow‐up, and assess cost‐effectiveness and relevant process measures that enable a deeper understanding of the mechanisms of effect. Natural experimental studies may also be useful to explore the effects of approaches not suited to RCT designs (e.g. to examine modifications to outdoor design). It is imperative that intervention studies include assessments of behavioural, health and educational outcomes where possible, thus speaking to policy makers in health and education sectors alike*.* Mixed‐methods studies are appropriate for developing a further understanding of what works, for whom and in what contexts.

## Conclusions

In order for the ‘school environment’ to be become a meaningful construct, it is important that researchers developing school‐based interventions recognize the importance and complexities of the environmental factors that can influence physical activity and sedentary behaviour. Approaches to increase physical activity or reduce sedentary behaviour within schools should address the multiple layers of school environment and how features of the school's physical, social and policy environment interact and influence each other to shape physical activity behaviours.

## Conflict of interest statement

No conflict of interest was declared

## Supporting information




**Table S1.** Search strategies.
**Table S2.** Quality assessment tool.
**Table S3.** Overview of quantitative studies.
**Table S4.** Overview of qualitative studies.

Supporting info item

## References

[1] Ekelund U , Luan J , Sherar LB , Esliger DW , Griew P , Cooper A . Moderate to vigorous physical activity and sedentary time and cardiometabolic risk factors in children and adolescents. JAMA 2012; 307: 704–712. 22337681 10.1001/jama.2012.156 PMC3793121

[2] Biddle SJH , Asare M . Physical activity and mental health in children and adolescents: a review of reviews. Br J Sports Med 2011; 45: 886–895. 21807669 10.1136/bjsports-2011-090185

[3] Singh A , Uijtdewilligen L , Twisk JWR , van Mechelen W , Chinapaw MJM . Physical activity and performance at school: a systematic review of the literature including a methodological quality assessment. Arch Pediatr Adolesc Med 2012; 166: 49–55. 22213750 10.1001/archpediatrics.2011.716

[4] Corder K , Sharp SJ , Atkin AJ *et al.* Change in objectively measured physical activity during the transition to adolescence. Br J Sports Med 2015; 49: 730–736. 24273308 10.1136/bjsports-2013-093190 PMC4453714

[5] Whitt‐Glover MC , Taylor WC , Floyd MF , Yore MM , Yancey AK , Matthews CE . Disparities in physical activity and sedentary behaviors among US children and adolescents: prevalence, correlates, and intervention implications. J Public Health Policy 2009; 30(Suppl 1): S309–S334. 19190581 10.1057/jphp.2008.46

[6] Brodersen NH , Steptoe A , Boniface DR , Wardle J . Trends in physical activity and sedentary behaviour in adolescence: ethnic and socioeconomic differences. Br J Sports Med 2007; 41: 140–144. 17178773 10.1136/bjsm.2006.031138 PMC2465219

[7] Dobbins M , Husson H , DeCorby K , LaRocca RL . School‐based physical activity programs for promoting physical activity and fitness in children and adolescents aged 6 to 18. Cochrane Database Syst Rev 2013; 2: CD007651. 10.1002/14651858.CD007651.pub2 PMC7197501 23450577

[8] Doak CM , Visscher TLS , Renders CM , Seidell JC . The prevention of overweight and obesity in children and adolescents: a review of interventions and programmes. Obes Rev 2006; 7: 111–136. 16436107 10.1111/j.1467-789X.2006.00234.x

[9] Marteau TM , Hollands GJ , Fletcher PC . Changing human behavior to prevent disease: the importance of targeting automatic processes. Science 2012; 337: 1492–1495. 22997327 10.1126/science.1226918

[10] Green LW , Richard L , Potvin L . Ecological foundations of health promotion. Am J Health Promot 1996; 10: 270–281. 10159708 10.4278/0890-1171-10.4.270

[11] Bonell C , Jamal F , Harden A *et al.* Systematic review of the effects of schools and school environment interventions on health: evidence mapping and synthesis. Public Health Res 2013; 1: 1. 25642578

[12] Langford R , Bonell C , Jones H *et al.* The World Health Organization's Health Promoting Schools framework: a Cochrane systematic review and meta‐analysis. BMC Public Health 2015; 15: 130. 25886385 10.1186/s12889-015-1360-y PMC4339015

[13] Langford R , Bonell C , Jones H , Campbell R . Obesity prevention and the Health Promoting Schools framework: essential components and barriers to success. Int J Behav Nutr Phys Act 2015; 12: 167. 10.1186/s12966-015-0167-7 PMC4330926 25885800

[14] Harrison F , Jones AP . A framework for understanding school based physical environmental influences on childhood obesity. Health Place 2012; 18: 639–648. 22281440 10.1016/j.healthplace.2011.12.009 PMC3759222

[15] Jago R , Baranowski T . Non‐curricular approaches for increasing physical activity in youth: a review. Prev Med 2004; 39: 157–163. 15207997 10.1016/j.ypmed.2004.01.014

[16] Lagarde F , LeBlanc C . Policy options to support physical activity in schools. Can J Public Health 2010; 101(Suppl): S9–S13. 21137137 10.1007/BF03405618 PMC6974204

[17] Escalante Y , García‐Hermoso A , Backx K , Saavedra JM . Playground designs to increase physical activity levels during school recess: a systematic review. Health Educ Behav 2014; 41: 138–144. 23836828 10.1177/1090198113490725

[18] Norris E , Shelton N , Dunsmuir S , Duke‐Williams O , Stamatakis E . Physically active lessons as physical activity and educational interventions: a systematic review of methods and results. Prev Med 2015; 72C: 116–125. 25562754 10.1016/j.ypmed.2014.12.027

[19] Bonell C , Wells H , Harden A *et al.* The effects on student health of interventions modifying the school environment: systematic review. J Epidemiol Community Health 2013; 67: 677–681. 23682106 10.1136/jech-2012-202247

[20] Robertson‐Wilson JE , Dargavel MD , Bryden PJ , Giles‐Corti B . Physical activity policies and legislation in schools: a systematic review. Am J Prev Med 2012; 43: 643–649. 23159260 10.1016/j.amepre.2012.08.022

[21] Pluye P , Gagnon M‐P , Griffiths F , Johnson‐Lafleur J . A scoring system for appraising mixed methods research, and concomitantly appraising qualitative, quantitative and mixed methods primary studies in mixed studies reviews. Int J Nurs Stud 2009; 46: 529–546. 19233357 10.1016/j.ijnurstu.2009.01.009

[22] Sallis JF , Prochaska JJ , Taylor WC . A review of correlates of physical activity of children and adolescents. Med Sci Sports Exerc 2000; 32: 963–975. 10795788 10.1097/00005768-200005000-00014

[23] Thomas J , Harden A , Oakley A *et al.* Integrating qualitative research with trials in systematic reviews. BMJ 2004; 328: 1010–1012. 15105329 10.1136/bmj.328.7446.1010 PMC404509

[24] Pluye P , Hong QN . Combining the power of stories and the power of numbers: mixed methods research and mixed studies reviews. Annu Rev Public Health 2014; 35: 29–45. 24188053 10.1146/annurev-publhealth-032013-182440

[25] Lonsdale C , Rosenkranz RR , Sanders T *et al.* A cluster randomized controlled trial of strategies to increase adolescents' physical activity and motivation in physical education: results of the Motivating Active Learning in Physical Education (MALP) trial. Prev Med 2013; 57: 696–702. 24035889 10.1016/j.ypmed.2013.09.003

[26] Dzewaltowski DA , Estabrooks PA , Welk G *et al.* Healthy youth places: a randomized controlled trial to determine the effectiveness of facilitating adult and youth leaders to promote physical activity and fruit and vegetable consumption in middle schools. Health Educ Behav 2009; 36: 583–600. 18469366 10.1177/1090198108314619 PMC2693233

[27] Knox GJ , Baker JS , Davies B *et al.* Effects of a novel school‐based cross‐curricular physical activity intervention on cardiovascular disease risk factors in 11‐ to 14‐year‐olds: the activity knowledge circuit. Am J Health Promot 2012; 27: 75–83. 23113776 10.4278/ajhp.110617-QUAN-258

[28] Cecchini JA , Fernandez‐Rio J , Mendez‐Gimenez A . Effects of Epstein's TARGET on adolescents' intentions to be physically active and leisure‐time physical activity. Health Educ Res 2014; 29: 485–490. 24650945 10.1093/her/cyu007

[29] Chatzisarantis NLD , Hagger MS . Effects of an intervention based on self‐determination theory on self‐reported leisure‐time physical activity participation. Psychol Health 2009; 24: 29–48. 20186638 10.1080/08870440701809533

[30] Gao Z , Lochbaum M , Podlog L . Self‐efficacy as a mediator of children's achievement motivation and in‐class physical activity. Percept Mot Skills 2011; 113: 969–981. 22403939 10.2466/06.11.25.PMS.113.6.969-981

[31] Cleland V , Dwyer T , Blizzard L , Venn A . The provision of compulsory school physical activity: associations with physical activity, fitness and overweight in childhood and twenty years later. Int J Behav Nutr Phys Act 2008; 5: 14. 18312621 10.1186/1479-5868-5-14 PMC2292742

[32] Bocarro JN , Kanters MA , Cerin E *et al.* School sport policy and school‐based physical activity environments and their association with observed physical activity in middle school children. Health Place 2012; 18: 31–38. 21900034 10.1016/j.healthplace.2011.08.007

[33] Cohen D , Scott M , Wang FZ , McKenzie TL , Porter D . School design and physical activity among middle school girls. J Phys Act Health 2008; 5: 719–731. 18820346 10.1123/jpah.5.5.719 PMC3689591

[34] Cradock AL , Melly SJ , Allen JG , Morris JS , Gortmaker SL . Characteristics of school campuses and physical activity among youth. Am J Prev Med 2007; 33: 106–113. 17673097 10.1016/j.amepre.2007.04.009 PMC2735893

[35] Lubans DR , Okely AD , Morgan PJ , Cotton W , Puglisi L , Miller J . Description and evaluation of a social cognitive model of physical activity behaviour tailored for adolescent girls. Health Educ Res 2012; 27: 115–128. 21680762 10.1093/her/cyr039

[36] McKenzie TL , Marshall SJ , Sallis JF , Conway TL . Student activity levels, lesson context, and teacher behavior during middle school physical education. Res Q Exerc Sport 2000; 71: 249–259. 10999262 10.1080/02701367.2000.10608905

[37] McKenzie TL , Prochaska JJ , Sallis JF , LaMaster KJ . Coeducational and single‐sex physical education in middle schools: impact on physical activity. Res Q Exerc Sport 2004; 75: 444–447. 10.1080/02701367.2004.10609179 15673045

[38] Goh YY , Bogart LM , Sipple‐Asher BK *et al.* Using community‐based participatory research to identify potential interventions to overcome barriers to adolescents' healthy eating and physical activity. J Behav Med 2009; 32: 491–502. 19544091 10.1007/s10865-009-9220-9 PMC2863037

[39] MacQuarrie C , Murnaghan D , MacLellan D . Physical activity in intermediate schools: the interplay of school culture, adolescent challenges, and athletic elitism. Qual Rep 2008; 13: 262–277.

[40] Azzarito L , Solmon MA , Harrison L . “… If I had a choice, I would …” A feminist poststructuralist perspective on girls in physical education. Res Q Exerc Sport 2006; 77: 222–239. 16898278 10.1080/02701367.2006.10599356

[41] Lamb P . Ritual associated with participation in physical education: the power of excuse notes. Eur Phys Educ Rev 2014; 20: 120–139.

[42] Shenton AK . Strategies for ensuring trustworthiness in qualitative research projects. Educ Inf 2004; 22: 63–75.

[43] Hobin EP , Leatherdale ST , Manske S , Dubin JA , Elliott S , Veugelers P . A multilevel examination of gender differences in the association between features of the school environment and physical activity among a sample of grades 9 to 12 students in Ontario, Canada. BMC Public Health 2012; 12: 74. 22272717 10.1186/1471-2458-12-74 PMC3330023

[44] Hobin E , Leatherdale S , Manske S , Dubin J , Elliott S , Veugelers P . A multilevel examination of factors of the school environment and time spent in moderate to vigorous physical activity among a sample of secondary school students in grades 9–12 in Ontario, Canada. Int J Public Health 2012; 57: 699–709. 22322666 10.1007/s00038-012-0336-2

[45] Boyle SE , Jones GL , Walters SJ . Physical activity among adolescents and barriers to delivering physical education in Cornwall and Lancashire, UK: a qualitative study of heads of PE and heads of schools. BMC Public Health 2008; 8: 273. 18673562 10.1186/1471-2458-8-273 PMC2518562

[46] Dagkas S , Stathi A . Exploring social and environmental factors affecting Adolescents' participation in physical activity. Eur Phys Educ Rev 2007; 13: 369–384.

[47] Hannay J , Dudley R , Milan S , Leibovitz PK . Combining Photovoice and focus groups: engaging Latina teens in community assessment. Am J Prev Med 2013; 44: S215–S224. 23415186 10.1016/j.amepre.2012.11.011

[48] Kirby J , Inchley J . Active travel to school: views of 10–13 year old schoolchildren in Scotland. Health Educ 2009; 109: 169–183.

[49] Kirby J , Levin KA , Inchley J . Socio‐environmental influences on physical activity among young people: a qualitative study. Health Educ Res 2013; 28: 954–969. 23969630 10.1093/her/cyt085

[50] Monge‐Rojas R , Garita‐Arce C , Sanchez‐Lopez M , Colon‐Ramos U . Barriers to and suggestions for a healthful, active lifestyle as perceived by rural and urban Costa Rican adolescents. J Nutr Educ Behav 2009; 41: 152–160. 19411048 10.1016/j.jneb.2008.03.002

[51] Hassandra M , Goudas M , Chroni S . Examining factors associated with intrinsic motivation in physical education: a qualitative approach. Psychol Sport Exerc 2003; 4: 211–223.

[52] Ntoumanis N , Pensgaard AM , Martin C , Pipe K . An idiographic analysis of amotivation in compulsory school physical education. J Sport Exerc Psychol 2004; 26: 197–214.

[53] Hyndman B , Telford A , Finch CF , Benson AC . Moving physical activity beyond the school classroom: a social‐ecological insight for teachers of the facilitators and barriers to students' non‐curricular physical activity. Aust J Teach Educ 2012; 37: 1–24.

[54] Bauer KW , Yang YW , Austin SB . “How can we stay healthy when you're throwing all of this in front of us?” – Findings from focus groups and interviews in middle schools on environmental influences on nutrition and physical activity. Health Educ Behav 2004; 31: 34–46. 14768656 10.1177/1090198103255372

[55] Kubik MY , Lytle L , Fulkerson JA . Fruits, vegetables, and football: findings from focus groups with alternative high school students regarding eating and physical activity. J Adolesc Health 2005; 36: 494–500. 15901514 10.1016/j.jadohealth.2004.05.010

[56] Booth ML , Wilkenfeld RL , Pagnini DL , Booth SL , King LA . Perceptions of adolescents on overweight and obesity: the weight of opinion study. J Paediatr Child Health 2008; 44: 248–252. 18194195 10.1111/j.1440-1754.2007.01267.x

[57] Dwyer JJ , Allison KR , Goldenberg ER , Fein AJ , Yoshida KK , Boutilier MA . Adolescent girls' perceived barriers to participation in physical activity. Adolescence 2006; 41: 75–89. 16689442

[58] Hohepa M , Schofield G , Kolt GS . Physical activity: what do high school students think? J Adolesc Health 2006; 39: 328–336. 16919793 10.1016/j.jadohealth.2005.12.024

[59] Robbins LB , Talley HC , Wu TY , Wilbur J . Sixth‐grade boys' perceived benefits of and barriers to physical activity and suggestions for increasing physical activity. J Sch Nurs 2010; 26: 65–77. 19850952 10.1177/1059840509351020

[60] Hohepa M , Scragg R , Schofield G , Kolt GS , Schaaf D . Social support for youth physical activity: importance of siblings, parents, friends and school support across a segmented school day. Int J Behav Nutr Phys Act 2007; 4: 54. 17996070 10.1186/1479-5868-4-54 PMC2186357

[61] Sallis JF , McKenzie TL , Conway TL *et al.* Environmental interventions for eating and physical activity – A randomized controlled trial in middle schools. Am J Prev Med 2003; 24: 209–217. 12657338 10.1016/s0749-3797(02)00646-3

[62] Ames C . Classrooms: goals, structures, and student motivation. J Educ Psychol 1992; 84: 261–271.

[63] Knowles AM , Niven A , Fawkner S . A qualitative examination of factors related to the decrease in physical activity behavior in adolescent girls during the transition from primary to secondary school. J Phys Act Health 2011; 8: 1084–1091. 22039126 10.1123/jpah.8.8.1084

[64] Slater A , Tiggemann M . “Uncool to do sport”: a focus group study of adolescent girls' reasons for withdrawing from physical activity. Psychol Sport Exerc 2010; 11: 619–626.

[65] Morton KL , Keith SE , Beauchamp MR . Transformational teaching and physical activity: a new paradigm for adolescent health promotion? J Health Psychol 2010; 15: 248–257. 20207668 10.1177/1359105309347586

[66] Smith MA , St. Pierre PE . Secondary students' perceptions of enjoyment in physical education: an American and English perspective. Phys Educ 2009; 66: 209–221.

[67] Constantinou P , Manson M , Silverman S . Female students' perceptions about gender‐role stereotypes and their influence on attitude toward physical education. Phys Educ 2009; 66: 85–96.

[68] Barr‐Anderson DJ , Young DR , Sallis JF *et al.* Structured physical activity and psychosocial correlates in middle‐school girls. Prev Med 2007; 44: 404–409. 17363050 10.1016/j.ypmed.2007.02.012

[69] Wenthe PJ , Janz KF , Levy SM . Gender similarities and differences in factors associated with adolescent moderate‐vigorous physical activity. Pediatr Exerc Sci 2009; 21: 291–304. 19827453 10.1123/pes.21.3.291 PMC2895819

[70] Utter J , Denny S , Robinson E , Ameratunga S , Milfont TL . Social and physical contexts of schools and neighborhoods: associations with physical activity among young people in New Zealand. Am J Public Health 2011; 101: 1690–1695. 21778475 10.2105/AJPH.2011.300171 PMC3154245

[71] Martin JJ , McCaughtry N , Flory S , Murphy A , Wisdom K . Using social cognitive theory to predict physical activity and fitness in underserved middle school children. Res Q Exerc Sport 2011; 82: 247–255. 21699104 10.1080/02701367.2011.10599752

[72] Hagger MS , Chatzisarantis NLD , Barkoukis V , Wang CKJ , Baranowski J . Perceived autonomy support in physical education and leisure‐time physical activity: a cross‐cultural evaluation of the trans‐contextual model. J Educ Psychol 2005; 97: 376–390.

[73] Jaakkola T , Washington T , Yli‐Piipari S . The association between motivation in school physical education and self‐reported physical activity during Finnish junior high school: a self‐determination theory approach. Eur Phys Educ Rev 2013; 19: 127–141.

[74] Jackson B , Whipp PR , Chua KL , Dimmock JA , Hagger MS . Students' tripartite efficacy beliefs in high school physical education: within‐ and cross‐domain relations with motivational processes and leisure‐time physical activity. J Sport Exerc Psychol 2013; 35: 72–84. 23404881 10.1123/jsep.35.1.72

[75] Moreno‐Murcia JA , Hernandez EH . The importance of supporting adolescents' autonomy in promoting physical‐sport exercise. Span J Psychol 2013; 16: E81. 24230944 10.1017/sjp.2013.81

[76] Zhang T , Solmon MA , Kosma M , Carson RL , Gu XL . Need support, need satisfaction, intrinsic motivation, and physical activity participation among middle school students. J Teach Phys Educ 2011; 30: 51–68.

[77] Slingerland M , Haerens L , Cardon G , Borghouts L . Differences in perceived competence and physical activity levels during single‐gender modified basketball game play in middle school physical education. Eur Phys Educ Rev 2013; 20: 20–35.

[78] Moore JB , Jilcott SB , Shores KA , Evenson KR , Brownson RC , Novick LF . A qualitative examination of perceived barriers and facilitators of physical activity for urban and rural youth. Health Educ Res 2010; 25: 355–367. 20167607 10.1093/her/cyq004 PMC10170971

[79] Tremblay MS , LeBlanc AG , Kho ME *et al.* Systematic review of sedentary behaviour and health indicators in school‐aged children and youth. Int J Behav Nutr Phys Act 2011; 8: 98. 21936895 10.1186/1479-5868-8-98 PMC3186735

[80] Brooke HL , Atkin AJ , Corder K , Ekelund U , van Sluijs EMF . Changes in time‐segment specific physical activity between ages 10 and 14 years: a longitudinal observational study. J Sci Med Sport 2014 DOI: 10.1016/j.jsams.2014.10.003. 10.1016/j.jsams.2014.10.003 PMC4678171 25459234

[81] Salmon J . Novel strategies to promote children's physical activities and reduce sedentary behavior. J Phys Act Health 2010; 7(Suppl 3): S299–S306. 21116014 10.1123/jpah.7.s3.s299

[82] Benden ME , Blake JJ , Wendel ML , Huber JC . The impact of stand‐biased desks in classrooms on calorie expenditure in children. Am J Public Health 2011; 101: 1433–1436. 21421945 10.2105/AJPH.2010.300072 PMC3134494

[83] Lanningham‐Foster L , Foster RC , McCrady SK *et al.* Changing the school environment to increase physical activity in children. Obesity 2008; 16: 1849–1853. 18535550 10.1038/oby.2008.282 PMC2690697

[84] Birnbaum AS , Evenson KR , Motl RW *et al.* Scale development for perceived school climate for girls' physical activity. Am J Health Behav 2005; 29: 250–257. 15899688 10.5993/ajhb.29.3.6 PMC2494732

[85] Button B , Trites S , Janssen I . Relations between the school physical environment and school social capital with student physical activity levels. BMC Public Health 2013; 13: 1191. 24341628 10.1186/1471-2458-13-1191 PMC3882326

[86] Durant N , Harris SK , Doyle S *et al.* Relation of school environment and policy to adolescent physical activity. J Sch Health 2009; 79: 153–159. 19292847 10.1111/j.1746-1561.2008.00384.x

[87] Fein AJ , Plotnikoff RC , Wild TC , Spence JC . Perceived environment and physical activity in youth. Int J Behav Med 2004; 11: 135–142. 15496341 10.1207/s15327558ijbm1103_2

[88] Fjørtoft I , Löfman O , Thorén KH . Schoolyard physical activity in 14‐year‐old adolescents assessed by mobile GPS and heart rate monitoring analysed by GIS. Scand J Public Health 2010; 38: 28–37. 21062837 10.1177/1403494810384909

[89] Galán I , Boix R , Medrano MJ , Ramos P , Rivera F , Moreno C . Individual factors and school‐based policies related to adherence to physical activity recommendations in Spanish adolescents. Prev Sci 2014; 15: 588–599. 23728581 10.1007/s11121-013-0407-5

[90] Graham DJ , Bauer KW , Friend S , Barr‐Anderson DJ , Nuemark‐Sztainer D . Personal, behavioral, and socio‐environmental correlates of physical activity among adolescent girls: cross‐sectional and longitudinal associations. J Phys Act Health 2014; 11: 51–61. 23250194 10.1123/jpah.2011-0239 PMC4107657

[91] Haerens L , Craeynest M , Deforche B , Maes L , Cardon G , De Bourdeaudhuij I . The contribution of home, neighbourhood and school environmental factors in explaining physical activity among adolescents. J Environ Public Health 2009 DOI: 10.1155/2009/320372. 10.1155/2009/320372 PMC2778568 20041023

[92] Haug E , Torsheim T , Samdal O . Physical environmental characteristics and individual interests as correlates of physical activity in Norwegian secondary schools: the health behaviour in school‐aged children study. Int J Behav Nutr Phys Act 2008; 5: 47. 10.1186/1479-5868-5-47 PMC2564975 18823545

[93] Haug E , Torsheim T , Sallis JF , Samdal O . The characteristics of the outdoor school environment associated with physical activity. Health Educ Res 2010; 25: 248–256. 18936270 10.1093/her/cyn050 PMC2839138

[94] Haug E , Torsheim T , Samdal O . Local school policies increase physical activity in Norwegian secondary schools. Health Promot Int 2010; 25: 63–72. 19884244 10.1093/heapro/dap040 PMC2824600

[95] Kanters MA , Bocarro JN , Edwards MB , Casper JM , Floyd MF . School sport participation under two school sport policies: comparisons by race/ethnicity, gender, and socioeconomic status. Ann Behav Med 2013; 45: S113–S121. 22993023 10.1007/s12160-012-9413-2

[96] Lee KS , Loprinzi PD , Trost SG . Determinants of Physical Activity in Singaporean Adolescents. Int J Behav Med 2011; 17: 279–286. 19760506 10.1007/s12529-009-9060-6

[97] Li M , Dibley MJ , Sibbritt D , Yan H . Factors associated with adolescents' physical inactivity in Xi'an City, China. Med Sci Sports Exerc 2006; 38: 2075–2085. 17146313 10.1249/01.mss.0000233802.54529.87

[98] Mandic S , Bengoechea EG , Stevens E , de la Barra SL , Skidmore P . Getting kids active by participating in sport and doing it more often: focusing on what matters. Int J Behav Nutr Phys Act 2012; 9: 86. 22788577 10.1186/1479-5868-9-86 PMC3416726

[99] Martensson F , Jansson M , Johansson M , Raustorp A , Kylin M , Boldermann C . The role of greenery for physical activity play at school grounds. Urban For Urban Green 2014; 13: 103–113.

[100] McLellan L , Rissel C , Donnelly N , Bauman A . Health behaviour and the school environment in New South Wales, Australia. Soc Sci Med 1999; 49: 611–619. 10452417 10.1016/s0277-9536(99)00136-7

[101] Millstein RA , Strobel J , Kerr J *et al.* Home, school, and neighborhood environment factors and youth physical activity. Pediatr Exerc Sci 2011; 23: 487–503. 22109776 10.1123/pes.23.4.487

[102] O'Malley PM , Johnston LD , Delva J , Terry‐McElrath YM . School physical activity environment related to student obesity and activity: a national study of schools and students. J Adolesc Health 2009; 45: S71–S81. 19699440 10.1016/j.jadohealth.2009.04.008

[103] Ridgers ND , Timperio A , Crawford D , Salmon J . What factors are associated with adolescents' school break time physical activity and sedentary time? PLoS One 2013; 8: e56838. 10.1371/journal.pone.0056838 PMC3572081 23418606

[104] Sallis JF , Conway TL , Prochaska JJ , McKenzie TL , Marshall SJ , Brown M . The association of school environments with youth physical activity. Am J Public Health 2001; 91: 618–620. 11291375 10.2105/ajph.91.4.618 PMC1446652

[105] Scott MM , Cohen DA , Evenson KR *et al.* Weekend schoolyard accessibility, physical activity, and obesity: The Trial of Activity in Adolescent Girls (TAAG) study. Prev Med 2007; 44: 398–403. 17292958 10.1016/j.ypmed.2006.12.010 PMC1978099

[106] Trang NH , Hong TK , Dibley MJ , Sibbritt DW . Factors associated with physical inactivity in adolescents in Ho Chi Minh City, Vietnam. Med Sci Sport Exerc 2009; 41: 1374–1383. 10.1249/MSS.0b013e31819c0dd3 19516164

[107] Yancey AK , Grant D , Kurosky S , Kravitz‐Wirtz N , Mistry R . Role modeling, risk, and resilience in California adolescents. J Adolesc Health 2011; 48: 36–43. 21185522 10.1016/j.jadohealth.2010.05.001

[108] Abarca‐Sos A , Bois JE , Zaragoza J , Generelo E , Julian JA . Ecological correlates of physical activity in youth: importance of parents, friends, physical education teachers and geographical localization. Int J Sport Psychol 2013; 44: 215–233.

[109] Bryan CL , Solmon MA . Student motivation in physical education and engagement in physical activity. J Sport Behav 2012; 35: 267–285.

[110] Cawley J , Meyerhoefer C , Newhouse D . The correlation of youth physical activity with state policies. Contemp Econ Policy 2007; 25: 506–517.

[111] Chow BC , McKenzie TL , Louie L . Physical activity and environmental influences during secondary school physical education. J Teach Phys Educ 2009; 28: 21–37.

[112] Jin J , Yun J . Three frameworks to predict physical activity behavior in middle school inclusive physical education: a multilevel analysis. Adapt Phys Act Q 2013; 30: 254–270. 10.1123/apaq.30.3.254 23860507

[113] Parish LE , Treasure DC . Physical activity and situational motivation in physical education: influence of the motivational climate and perceived ability. Res Q Exerc Sport 2003; 74: 173–182. 12848230 10.1080/02701367.2003.10609079

[114] Zhang T , Solmon MA , Gao Z , Kosma M . Promoting school students' physical activity: a social ecological perspective. J Appl Sport Psychol 2012; 24: 92–105.

[115] Fuller D , Sabiston C , Karp I , Barnett T , O'Loughlin J . School sports opportunities influence physical activity in secondary school and beyond. J Sch Health 2011; 81: 449–454. 21740429 10.1111/j.1746-1561.2011.00613.x

[116] Barkoukis V , Hagger MS . The trans‐contextual model: perceived learning and performance motivational climates as analogues of perceived autonomy support. Eur J Psychol Educ 2013; 28: 353–372.

[117] Beauchamp MR , Liu Y , Morton KL *et al.* Transformational teaching and adolescent physical activity: multilevel and mediational effects. Int J Behav Med 2014; 21: 537–546. 23760732 10.1007/s12529-013-9321-2

[118] Hagger MS , Chatzisarantis NLD , Culverhouse T , Biddle SJH . The processes by which perceived autonomy support in physical education promotes leisure‐time physical activity intentions and behavior: a trans‐contextual model. J Educ Psychol 2003; 95: 784–795.

[119] Pihu M , Hein V , Koka A , Hagger MS . How students' perceptions of teachers' autonomy‐supportive behaviours affect physical activity behaviour: an application of the trans‐contextual model. Eur J Sport Sci 2008; 8: 193–204.

[120] Mckenzie TL , Sallis JF , Prochaska JJ , Conway TL , Marshall SJ , Rosengard P . Evaluation of a two‐year middle‐school physical education intervention: M‐SPAN. Med Sci Sport Exerc 2004; 36: 1382–1388. 10.1249/01.mss.0000135792.20358.4d 15292747

[121] How YM , Whipp P , Dimmock J , Jackson B . The effects of choice on autonomous motivation, perceived autonomy support, and physical activity levels in high school physical education. J Teach Phys Educ 2013; 32: 131–148.

[122] Perlman D . The influence of the social context on students in‐class physical activity. J Teach Phys Educ 2013; 32: 46–60.

[123] Schuldheisz JM , van der Mars H . Active supervision and students' physical activity in middle school physical education. J Teach Phys Educ 2001; 21: 75–90.

[124] Hobin EP , Leatherdale S , Manske S , Dubin JA , Elliott S , Veugelers P . Are environmental influences on physical activity distinct for urban, suburban, and rural schools? A multilevel study among secondary school students in Ontario, Canada. J Sch Health 2013; 83: 357–367. 23517004 10.1111/josh.12039

