# Supplementary material for: The school environment and adolescent physical activity and sedentary behaviour: a mixed‐studies systematic review
Source: Obes Rev. 2015 Dec 18;17(2):142–58. doi: 10.1111/obr.12352 (PMC4914929; doi:10.1111/obr.12352)
Supplement: Supplementary file 1 — Supporting info item [file OBR-17-142-s001.docx]

**Online Supplemental Material:**

**The school environment and adolescent physical activity and sedentary behaviour: A mixed studies systematic review**

Katie L Morton^1*^, Andrew J Atkin^1^, Kirsten Corder^1^, Marc Suhrcke^2^, Esther MF van Sluijs^1^

^1^ MRC Epidemiology Unit and UKCRC Centre for Diet and Activity Research (CEDAR), University of Cambridge

^2^ Centre for Health Economics, University of York

* Corresponding author: Katie Morton

Address: UKCRC Centre for Diet and Activity Research (CEDAR), MRC Epidemiology Unit, University of Cambridge School of Clinical Medicine, Box 285 Institute of Metabolic Science, Cambridge Biomedical Campus, Cambridge, UK, CB2 0QQ

Email: [km576@medschl.cam.ac.uk](mailto:km576@medschl.cam.ac.uk)

**Table S1. Search strategies**

| **Search strategy for Medline**  ("physical activity"[Title/Abstract]) OR "physical activities"[Title/Abstract]) OR exercise[MeSH]) OR "sedentary time"[Title/Abstract]) OR "sedentary behaviour"[Title/Abstract]) OR "sedentary behavior"[Title/Abstract]) OR "active transport"[Title/Abstract]) OR "active travel"[Title/Abstract]) OR cycling[Title/Abstract]) OR walking[Title/Abstract]) OR sport[Title/Abstract]) OR sports[Title/Abstract]) OR fitness[Title/Abstract]) OR "energy expenditure"[Title/Abstract]) OR sitting[Title/Abstract]) OR "screen time"[Title/Abstract]))  AND  ((school*[Title/Abstract]) OR teacher*[Title/Abstract]))  AND  (policy[Title/Abstract]) OR policies[Title/Abstract]) OR environment*[Title/Abstract]) OR climate*[Title/Abstract]) OR curriculum*[Title/Abstract]) OR classroom*[Title/Abstract]) OR "green space"[Title/Abstract]) OR "green spaces"[Title/Abstract]) OR teaching[Title/Abstract]) OR playground*[Title/Abstract]) OR playtime[Title/Abstract]) OR recess[Title/Abstract])) |
| --- |
| **Search strategy for Web of Science**  TOPIC: ("physical* activ*") OR TOPIC: ("sedentary time") OR TOPIC: ("sedentary behavi*") OR TOPIC: ("active transport*") OR TOPIC: ("active travel*") OR TOPIC: (cycling) OR TOPIC: (walking) OR TOPIC: (sport*) OR TOPIC: (fitness) OR TOPIC: ("energy expenditure") OR TOPIC: (sitting) OR TOPIC: ("screen time")  AND  TOPIC: (school*) OR TOPIC: (teacher*)  Timespan=All years  Search language=English  AND  TOPIC: (policy) OR TOPIC: (policies) OR TOPIC: (environment*) OR TOPIC: (climate*) OR TOPIC: (curriculum*) OR TOPIC: (classroom*) OR TOPIC: ("green space") OR TOPIC: ("green spaces") OR TOPIC: (teaching) OR TOPIC: (playground*) OR TOPIC: (playtime) OR TOPIC: (recess)  Timespan=All years  Search language=English |
| **Search strategy for Psych INFO**  (( TI policy OR AB policy OR KW policy ) OR ( TI policies OR AB policies OR KW policies ) OR ( TI environment* OR AB environment* OR KW environment* ) OR ( TI climate* OR AB climate* OR KW climate* ) OR ( TI curriculum* OR AB curriculum* OR KW curriculum* ) OR ( TI classroom* OR AB classroom* OR KW classroom* ) OR ( TI "green space*" OR AB "green space*" OR KW "green space*" ) OR ( TI teaching* OR AB teaching* OR KW teaching* ) OR ( TI playground* OR AB playground* OR KW playground* ) OR ( TI playtime* OR AB playtime* OR KW playtime* OR TI recess OR AB recess OR KW recess ))    (( TI school* OR AB school* OR KW school* ) OR ( TI teacher* OR AB teacher* OR AB teacher* ))  (( TI "physical* activ*" OR AB "physical* activ*" OR KW "physical activ*" ) OR ( TI "sedentary time" OR AB "sedentary time" OR KW "sedentary time" ) OR ( TI "sedentary behav*" OR AB "sedentary behav*" OR KW "sedentary behav*" ) OR ( TI "active travel*" OR AB "active travel*" OR KW "active travel*" ) OR ( TI "active transport*" OR AB "active transport*" OR KW "active transport*" ) OR ( TI walking OR AB walking OR KW walking ) OR ( TI cycling OR AB cycling OR KW cycling ) OR ( TI sport* or AB sport* OR KW sport* ) OR ( KW exercise ) OR ( TI sitting OR AB sitting OR KW sitting ) OR ( TI "screen time" OR AB "screen time" OR KW "screen time" )) |
| **Search strategy for ProQuest**  *ERIC; Australian Education Index; British Education Index; Physical Education Index; ProQuest Dissertations & Theses A&I*  su(exercise) OR ab("physical* activ*") OR ti("physical* activ*") OR ab("sedentary time") OR ti("sedentary time") OR ab(sport) OR ti(sport) OR ab("sedentary behav*") OR ti("sedentary behav*") OR ab("active travel") OR ti("active travel*") OR ab("active transport*") OR ti("active transport*") OR ab(cycling) OR ti(cycling) OR ab(walking) OR ti(walking) OR ab("energy expenditure") OR ti ("energy expenditure") OR ab(fitness) OR ti(fitness) OR ab(sitting) OR ti(sitting) OR ab("screen time") OR ti("screen time")  AND  ab(school*) OR ti(school*) OR ab(teacher*) OR ti(teacher*)  AND  ab(policy) OR ti(policy) OR ab(policies) OR ti(policies) OR ab(environment*) OR ti(environment*) OR ab(climate*) OR ti(climate*) OR ab(curriculum*) OR ti(curriculum*) OR ab(classroom*) OR ti(classroom*) OR ab("green space*") OR ti("green space*") OR ab(teaching) OR ti(teaching) OR ab(playground*) OR ti(playground*) OR ab(playtime*) OR ti(playtime*) OR ab(recess) OR ti(recess)  Filters: English |

**Table S2. Quality assessment tool**

| **Types of mixed methods study components or primary studies in a SMSR context** | **Methodological quality criteria** |
| --- | --- |
| 1. Qualitative | Qualitative objective or question |
|  | Appropriate qualitative approach or design or method |
|  | Description of the context |
|  | Description of participants and justification of sampling |
|  | Description of qualitative data collection and analysis |
|  | Discussion of researchers’ reflexivity |
| 0-1 = * 2-3 = ** 4-5 = *** 6 = **** | |
| 2. Quantitative experimental | Appropriate sequence generation and/or randomization |
|  | Allocation concealment and/or blinding |
|  | Complete outcome data and/or low withdrawal/drop-out |
|  | Appropriate outcome measure (PA/DES) |
| 3. Quantitative observational | Appropriate sampling and sample |
|  | Justification of measurements (validity and standards) - outcome (PA/SED); prospective if possible |
|  | Justification of measurements (validity and standards) - exposure (validated tool/objective measures if possible) |
|  | Control of confounding variables |
| 1=* 2= ** 3= *** 4=**** |  |

Sections in red font are the modified items

**Table S3. Overview of quantitative studies**

|  | **Study ID and country** | **Sample** | **Intervention/ Exposure variable** | | **Outcome measures** | **Key findings** |
| --- | --- | --- | --- | --- | --- | --- |
|  | *Whole-school Environment* | | | | | |
| 68 | Barr-Anderson et al (2007)  USA  O (CS)  ** | 2791 6^th^ grade female adolescents from 6 middle schools | **Social Environment:** School PA Climate  Girls perceptions of the influence of teachers and boys on the school climate for girls to be physically active | | Self-reported **involvement in structured activities** (inside and outside school) | - No association between school climate and participation in structured activities. |
| 84 | Birnbaum et al (2005)  USA  O (CS)  ** | 605 6^th^ grade (mean age = 11.5 years) and 8^th^ grade (mean age 13.3 years) female adolescents from 6 middle schools | **Social Environment:** School PA Climate  Girls perceptions of the influence of teachers and boys on the school climate for girls to be physically active | | Physical activity questionnaire for older children (PAQ-C); **Total MVPA** (PE, lunch, after-school, evenings and weekend) | - Positive direct association of the ‘teacher’ factor, but not the ‘boy’ factor, with girls' self-reported physical activity. |
| 32 | Bocarro et al (2012)  USA  O (CS)  **** | 6735 adolescents were observed from 4 middle schools (2 schools with intramural policies and 2 schools with inter-scholastic policies | **Physical, Social and Policy environment:**  Intramural versus varsity (inter-scholastic) sport policy; activity setting (type, location, size and boundaries); level of adult supervision | | System for Observing Play and Leisure in youth (SOPLAY); **after-school MVPA** (in specific areas) | - Odds of engaging in higher levels of PA lower in boys attending varsity school than intramural school (non-significant for girls) - No effects of supervision type on PA - Activity setting positively associated with odds of engaging in activity in boys but not girls. - Athletic facilities were under-utilized in all schools |
| 85 | Button et al (2013)  Canada  O(CS)  *** | 18,875 adolescents in grades 6-10 from 331 schools | **Physical and Social environment:**  Access to facilities and school social capital (school climate: social cohesion) | | WHO HBSC questionnaire; **MVPA at school** (in class and free time at school) | - School physical environment (access to facilities) and social environment (social capital) were positively associated with MVPA at school. |
| 31 | Cleland et al (2008)  Australia  O(CS &P)  *** | 6412 adolescents aged 9-15 from 109 schools (elementary and secondary) | **Policy environment:**  Duration of compulsory PE and school sport | | Baseline: Self-reported past week duration and frequency of PA (PE and sport) and non-school PA; **total PA and school PA**  Follow up: **pedometer steps** and International Physical Activity Questionnaire (IPAQ); **total PA and LTPA** | - At baseline and follow-up, median total physical activity was similar in participants who attended low, medium and high physical activity schools - There was no difference in the prevalence of high total physical activity in childhood or adulthood across compulsory school physical activity categories. |
| 33 | Cohen et al (2008)  USA  O(CS)  **** | 1566 grade 6 adolescent girls (mean age = 11.8 years) from 36 middle schools | **Physical environment:** Building footprint and number of active outdoor amenities | | Accelerometer assessed (Actigraph); **total within- school activity** (MVPA and LPA) | - Positive association between outdoor facilities and MVPA. - No association between building footprint and LPA or MVPA. |
| 34 | Cradock et al (2007)  USA  O(CS)  **** | 248 adolescents in grades 7-8 (mean age = 13.7 years) from 10 middle schools | **Physical environment:** School building and campus characteristics | | Accelerometer (Tritrac- RT3); **within-school PA** (movement) | - Positive associations between school campus area (per student), building area (per student), and play area (per student) and PA. |
| 86 | Durant et al (2009)  USA  O(CS)  ** | 165 adolescents aged 12-18 (mean age = 14.6 years) from schools in 3 cities in the USA | **Policy and Physical environment:**  Days of PE; sports equipment accessibility; after-school field access: supervised after school PA | | Self-reported **PA at school recreation facilities**, **overall PA** (MVPA) and **TV watching** (time) during typical school week | - Positive association between days of PE and PA - The association between after-school field access and overall PA was mediated by use of publicly accessible school facilities for PA. - After-school supervised PA and school PA equipment were not associated with overall PA. - No association between any school factor and TV watching |
| 26 | Dzewaltowski et al (2009)  USA  E(cRCT)  *** | 1582 adolescents in grade 6-8 (mean age = 12.36 years in intervention and 12.4 years in control) in 16 middle schools | **Social and Policy environment:**  Development of personal and proxy agency of adult leaders and youth to build school environments to promote PA: Training for school coordinators; change teams; facilitate student leadership; after-school program to implement environmental changes | | Previous day PA recall; (PDPAR); **After-school PA** (VPA and MVPA) | - After intervention, intervention schools significantly changed in PA compared to control schools. - Proxy efficacy to influence school PA environments mediated the program effects |
| 87 | Fein et al (2004)  Canada  O(CS)  ** | 610 adolescents in grades 9-12 from 4 secondary schools | **Physical environment:**  Physical environmental resources (gym space; sport/exercise equipment and athletic facilities) | | Energy expenditure based on the Leisure Time Exercise questionnaire (LTEQ); **total energy expenditure** (moderate and hard intensity activities) | - After controlling for other factors, perceived *availability* of resources was not a significant predictor of PA - Percevied *importance* of resources was a significant predictor of PA. |
| 88 | Fjortoft et al (2010)  Norway  O(CS)  *** | 81 9^th^ grade adolescents (mean age = 14 years) from 2 schools | **Physical environment:**  Schoolyard environment | | **Lunch time activity pattern and intensity**; GPS and heart rate: Combined movement and heart rate used as a proxy for PA | - Low activity levels dominated in both schools (no gender differences) - Activities in the handball area showed the most activity (higher intensity in girls than boys) - Except for handball goal area, available facilities and surrounding fields were not extensively used by 9^th^ graders during breaks. |
| 115 | Fuller et al (2011)  Canada  O(P)  **** | 808 adolescents (mean age = 12.7 years) from 10 secondary schools | **Policy environment:**  Number of intramural and extramural (interscholastic) sports opportunities | | 7-day recall adapted from the Weekly Activity Checklist; **moderate, vigorous and total PA number of PA sessions per week** (outside of PE), | - Controlling for multiple demographic factors, adolescents in high intramural schools reported more total PA and vigorous PA than adolescents in low intramural schools - Provision of extramural sport was not associated with adolescents’ physical activity. |
| 89 | Galan et al (2014)  Spain  O(CS)  *** | 15,902 adolescents aged 11-18 from 328 secondary schools | **Policy environment:**  *School-based policies for PA:* Participation of school in PA programmes; training teachers about PA; written guidelines about PA; Number of compulsory PE classes; perceived sustainability of facilities for PA; organisation of PA outside of school. | | Number of days exceeding 60 minutes of MVPA over last 7 days; **overall PA (adherence to guidelines)** | - Controlling for family and personal factors, students from schools with high level of policies to promote PA were more likely to meet PA recommendations. |
| 90 | Graham et al (2014)  USA  O(CS & P)  *** | 356 female adolescents (mean age 15.8 years) from 12 secondary schools | **Social environment:**  Teacher (and other staff) support (encouragement) for PA | | Three-Day Physical Activity Recall (3dPAR); **total PA and MVPA** | - Positive cross-sectional association between teacher support and total PA. - No longitudinal association between change in teacher support and either PA outcome. |
| 91 | Haerens et al (2009)  Belgium  O(CS)  *** | 523 adolescents in 7^th^-8^th^ grade (mean age 12.7 years) from 4 middle schools | **Physical, Social and Policy environment:**  Accessibility of sport facilities and sports materials; availability of supervision and extracurricular activities (during breaks and after school hours) | | Flemish PA questionnaire (FPAQ): **Participation in leisure time activities and extracurricular sport**; a subsample wore an accelerometer for 6 days to measure **total MVPA** | - Availability of (organised) extra physical activities was positively associated with participation in extracurricular PA participation (boys only) - Provision of supervised activity was positively associated with participation in extracurricular PA participation (girls only) - For boys, significant interactions with perceived benefits were found, with stronger correlations between supervision and engagement in extracurricular activities among boys reporting more perceived benefits of PA. - For girls, significant interactions with perceived benefits were found, with stronger correlations between organized activities and engagement in extracurricular activities among girls reporting more perceived benefits of PA. |
| 92 | Haug et al (2008)  Norway  O(CS)  *** | 1347 adolescents in grade 8 (mean age = 13 years) from 68 secondary schools | **Physical environment:**  Physical environment characteristics (facilities) | | Self-reported **PA during recess** (MVPA and VPA) | - Students attending schools with many facilities were more likely to be physically active than students in schools with lower facilities - Open fields, outdoor obstacle courses, playground equipment and a room with cardio and weightlifting equipment were associated with increased participation in PA. - The interaction term demonstrated that students' interests (interested in being more physically active) might moderate the effect of facilities on recess PA. |
| 94 | Haug et al (2010)  Norway  O(CS)  *** | 1347 adolescents in grade 8 (mean age = 13 years) from 68 secondary schools | **Policy environment:**  PE class policy and extra-curricular PE policy; PA policy (written); organised PA in non-curricular time (e.g., lunch, after school) | | Self-reported **PA during recess** (MVPA and VPA) | - Schools with a written policy for PA and schools that frequently organize physical activity during non-curricular school time had a higher proportion of students reporting recess physical activity. - Although not significant, providing PE classes five times a week were both associated with a higher proportion of students being active during recess. |
| 93 | Haug et al (2010)  Norway  O(CS)  *** | 6858 adolescents aged 13-15 in grades 8-10 from 21 secondary schools and 29 combined schools | **Physical environment:**  Physical environment characteristics (facilities) | | Self-reported **PA during recess** (MVPA and VPA) | - Students with a larger number of outdoor facilities were more likely to be active during recess compared with students with fewer facilities - Four of 8 characteristics (soccer fields, areas for hopscotch/skipping rope, playground equipment and sledding hill) were associated with PA in boys - One characteristic (sledding hill) was a predictor for girls - Outcomes not moderated by recess time. |
| 44 | Hobin et al (2012)  Canada  O(CS)  *** | 22117 adolescents in grades 9-12 from 76 secondary schools | **Policy and Physical environment:**  School PE and PA policy (intramural versus varsity) and PA facilities | | **Total MVPA** (average daily minutes) | - Students attending a school that offered daily physical education or provided an alternate room for physical activity spent more time in MVPA than students attending a school without these resources |
| 43 | Hobin et al (2012)  Canada  O(CS)  *** | 21754 adolescents in grades 9-12 from 76 secondary schools | **Policy and Physical environment:**  School PE and PA policy (intramural versus varsity) and PA facilities | | **Total MVPA** (average daily minutes) | - Positive association between daily PE and MVPA - Positive association between intramural activities and MVPA (girls only) - No association for varsity sport and MVPA - Attending a school with an ‘alternate room’ for PA was associated with greater PA for girls and boys - No association for any other facilities. |
| 124 | Hobin et al (2013)  Canada  O(CS)  *** | 22117 adolescents in grades 9-12 from 76 secondary schools | **Policy and Physical environment:**  School PE and PA policy (intramural versus varsity) and PA facilities | | **Total MVPA** (average daily minutes) | - None of the environment-level factors were associated with time spent in PA in rural schools. - Having an alternate room for PA associated with greater PA in urban schools. - Offering daily PE and having an alternate room for PA associated with greater activity in suburban schools. |
| 60 | Hohepa et al (2007)  New Zealand  O(CS)  *** | 3471 adolescents aged 12-18 (junior high school: years 7-11; senior high school: year 12-13) from 7 secondary schools | **Social environment:**  School encouragement for PA | | Self-reported **active transportation**; **PA during lunchtime** and **after-school PA** (NZ Child Nutrition Survey, based on the PAQ-C); classified as ‘active’ or ‘less active’ for each item. | - Positive (univariate) association between school support (encouragement) and lunch-time PA for junior high and senior high students - After controlling for gender/ethnicity and other predictors, school encouragement not a significant predictor of after-school PA - After controlling for gender/ethnicity and other predictors, school encouragement only a significant predictor of lunch-time PA for junior high students. |
| 95 | Kanters et al (2013)  O(CS)  USA  *** | 2582 adolescents in grades 6-8 (aged 11-14) from 4 middle schools | **Policy environment:**  Intramural versus interscholastic sports policy | | Self-reported **school sports participation** (students asked to identify school sports in which they had participated during the school year). | - Students at school with intramural policies participated in more school sports - Boys were more likely to participate in after-school sports at intra-mural schools - Low-income and black children had greater participation in intramural programs compared to interscholastic programs. |
| 27 | Knox et al (2012)  UK (Wales)  QuE  ** | 155 (I) and 77(C) adolescents in grade 8 (mean age =12.4 years) from 1 secondary school | **Policy environment:**  Active lesson content (implementing typical classroom tasks during brisk walking) | | Physical activity questionnaire for adolescents (PAQ-A); **total PA** | - No change in PA either group - Significant improvements in high density lipoprotein cholesterol to total cholesterol ratio and glucose were evident for the intervention group. |
| 96 | Lee et al (2011)  Singapore  O(CS)  *** | 1814 adolescents aged 13-16 (mean age = 14.4 years) from 6 secondary schools | **Policy environment:**  School activity program (availability of supervised school activity programmes) | | Three-day physical activity recall (3DPAR); **total PA** (mean energy expenditure; METs) | - No association between school activity programs and METs once added into a full model with other demographic and psycho-social predictors. |
| 97 | Li et al (2006)  China  O(CS)  *** | 1787 adolescents aged 11-17 from 30 junior high schools | **Physical and Policy environment:**  Availability of playgrounds, ovals, gyms, sports equipment, schedules for sports meetings, morning exercises, class recess exercise | | Adolescent PA recall questionnaire (ADPAR) adapted to Chinese adolescent; **total** **physical inactivity** | - For boys, lack of class recess sports and infrequent sports meetings were associated with inactivity; boys attending schools that did not permit bike riding were less likely to be inactive. - For girls, having fewer sports meetings and more sessions of PE per week were associated with inactivity. |
| 35 | Lubans et al (2012)  Australia  O(CS)  **** | 1518 grade 8 females (mean age = 13.6 years) from 24 secondary schools | **Physical environment:**  Facilities (perceived quality, accessibility and availability) | | Actigraph; **total PA and MVPA** (counts per minute) | - Positive association between the school physical environment and total PA but not MVPA - Positive association between school environment and self-efficacy for PA - No association between school environment and behavioural strategies for PA |
| 98 | Mandic et al (2012)  New Zealand  O(CS)  *** | 1837 adolescents in grades 9-13 (mean age = 14.6 years) from 19 secondary schools | **Policy and physical environment:**  School sport resources (participants asked to select up to 5 sports they participate in and asked about availability, competitions, coaching and management at school) | | Self-reported **in school sport and out of school sport**. | - Controlling for other factors, the quality of sport management is positively associated with time spent participating in sport. - Controlling for other factors, sufficient sports grounds and courts at school is negatively associated with time spent participating in sports. |
| 99 | Martensson et al (2014)  Sweden  O(CS)  *** | 97 adolescents in grade 6 (aged 12-13) from 1 ‘grey’ school and 1 ‘green’ school | **Physical environment:**  School design (greenery) | | Pedometer steps; **within-school PA** | - No association of school greenery and PA. |
| 71 | Martin et al (2011)  USA  O(CS)  ** | 506 adolescents in grades 6-7 (aged 10-14; mean age = 12 years) from 5 middle schools | **Physical and Social environment:**  Physical subscale: how physically ‘friendly’ the school is; equipment, facilities and programming.  Social subscale: Teacher values and supervision | | Godin Leisure-Time Exercise Questionnaire (GLTEQ); **total free-time PA** (metabolic equivalents) | - Significant negative correlation between school PA environment score and PA - Regression analysis revealed no association between school environment (physical or social) and PA. |
| 100 | McLellan et al (1999)  Australia  O(CS)  ** | 3998 adolescents in grades 6, 8 and 10 from 115 schools (mixture of secondary and elementary schools) | **Social environment:**  School support and teacher support | | PA survey (adapted from the European HSBC survey); **Outside of school PA** dichotomized into ‘healthy’ or non-healthy’ (usually exercised in free time or not) | - No association between school support and PA. - Teacher support for PA positively associated with PA. |
| 101 | Millstein et al (2011)  USA  O(CS)  ** | 137 adolescent aged 12-18 (mean age = 14.6 years) | **Physical environment:**  Equipment: availability of school PA spaces and equipment | | Self-reported **overall PA**; active transports; participation in organised PA outside of school; School PA and PE | - Positive association between number of school PA equipment pieces and PA. |
| 102 | O’Malley et al (2009)  USA  O(CS)  *** | Data from 2 studies included approximately 45,000 students in grades 8-12 from 410 secondary schools | **Policy environment:**  PE requirements and PE class duration and fitness testing policies(provides fitness testing/ provides parents with results of fitness testing) | | Three questions assessing: **participation in sport/ athletics/ exercising**, **vigorous exercise**, and **participation in school athletic (sport) teams** (dichotomized into ‘less than almost every day’ and ‘almost every day’) | - After controlling for key demographic variables, PE requirement associated with lower odds of exercising vigorously nearly every day/every day (grade 8 only). - Longer PE classes associated with lower odds of being active daily/almost daily (grade 8-10 only) and participating in less school athletics (grade 12 only). - School fitness testing associated with less odds of participating in school athletic teams (grade 8 only). No associations with fitness testing policies and any other PA outcome or in any other grade. |
| 103 | Ridgers et al (2013)  Australia  O(CS)^ᴛ^  **** | 146 adolescents* (mean age = 14.1 years) | **Policy environment:**  Lunch and break time length | | Actigraph: t**ime spent in sedentary, light and MVPA at break times** | - No association of break time length with sedentary time or MVPA |
| 104 | Sallis et al (2001)  USA  O(CS)  *** | Students from 24 middle schools (mean enrollment = 1081 students) in grades 8-6 | **Physical and Social environment:**  Area type, area size and permanent improvements, presence of equipment, level of supervision. | | System for Observing Play and Leisure in youth (SOPLAY); **within-school (free-time) MVPA** (in specific areas) | - Positive association for area size, area type and permanent improvements and PA - No association between level of supervision and PA - Interaction effects: More girls active when equipment is not present in indoor area, however equipment enhanced outdoor activity - Boys most active on courts with high supervision (when equipment is available) - Boys demonstrate more PA with highest levels of permanent improvements and supervision. |
| 61 | Sallis et al (2003)  USA  E(RCT)  *** | Students from 24 middle schools (mean enrollment = 1109) in grades 6-8 | **Physical, Social and Policy environment:**  To increase PA in PE: changing lesson context, structure and teaching behaviour.  To increase PA on campus during leisure periods throughout school day: increasing supervision, equipment and organised activities | | **Within-school MVPA** (energy expenditure); System for Observing Fitness Instruction Time (SOFIT) for **PE class PA** and  System for Observing Play and Leisure in youth (SOPLAY) for **within-school (free-time) PA** | - Significant intervention effect for PA for total group and boys, but not for girls. |
| 105 | Scott et al (2007)  USA  O(CS)  **** | 1556 female adolescents in grade 6 from middle schools | **Policy environment:**  School amenities and accessibility during weekends | | Accelerometers (Actigraph) assessed **total weekend MVPA** | - No association between school amenities accessibility (at weekend) and weekend PA. |
| 106 | Trang et al (2009)  Vietnam  O(CS)  *** | 2684 adolescents in grades 6-9 from 31 junior high schools | **Policy and Physical environment:**  PE sessons per week, morning recess exercises | | Adolescent PA recall questionnaire (APARQ); **total PA** (divided into 2 groups ‘physically active’ and ‘insufficiently active’ | - Participants at schools without exercises during recess were more likely to be inactive - Adolescents at schools having one or two sports meetings per year were less likely to be inactive - More frequent PE also associated with reduced odds of inactivity in girls. |
| 70 | Utter et al (2011)  New Zealand  O(CS)  ** | 9107 adolescents aged 13-17 from 96 secondary schools | **Social environment:**  School encouragement for PA | | Single item: **overall PA** (days of MVPA over last week) | - No association between school encouragement and PA. |
| 69 | Wenthe et al (2009)  USA  O(CS)  ** | 205 adolescents aged 12-14 (mean age = 13 years) | **Social environment:**  School climate (boys PA and teacher PA climate) | | Physical activity questionnaire for adolescents (PAQ-A); **total MVPA**  Accelerometer (Actigraph) assessed **total MVPA** | - No association between school climate and PA (self-report or objectively measured). |
| 107 | Yancey et al (2011)  USA  O(CS)  ** | 4010 adolescents aged 12-17 (mean age = 14.5) | **Social environment:**  School connectedness: students asked about adults at school who cared about them | | Self-reported *regular* **total MVPA** (at least 20 minutes of moderate or vigorous exercise on 3 or more of the past 7 days); California Health Interview Survey | - School connectedness positively associated with PA. |
|  | *PE environment only* | | | | | |
| 108 | Abarca-Sos et al (2013)  Spain  O(CS)  *** | 1272 adolescents aged 13-15 (mean age 13.5 years) from public and private secondary schools | **Social environment:** Teaching behaviours  PE teacher social influence (role modelling, social support and social influence) | Assessment of PE levels questionnaire; involvement in organised and non-organised PA and sport and PE summed to create PA index (**total PA**) | | - Significant negative correlation between PE teacher influence and adolescent PA. - Structural equation modelling demonstrated that the effects of PE teacher influence were mediated by perceived competence and value. There was a negative relationship between PE teacher behaviours and adolescent perceived competence and values. |
| 116 | Barkoukis et al (2013)  Greece  O(P)  *** | 170 adolescents (mean age 16.9 years) in 3 secondary schools | **Social environment:** Motivational climate  Perceived autonomy support and perceptions of learning and performance climates | Leisure Time Exercise Questionnaire (LTEQ); **vigorous leisure time PA** | | - No direct association (correlation) between perceived autonomy support and PA. - No direct association (correlation) between motivational climate and PA. - The results of the path analyses indicated that perceived learning climate predicted autonomous motivation and mediated perceived autonomy support on autonomous motivation in PE. Results also confirmed the mediating role of the TPB variables for adolescent leisure time-PA. |
| 117 | Beauchamp et al (2014)  Canada  O(P)  *** | 2948 adolescents in grades 8-10 (means age = 14.3 years) from 6 secondary schools | **Social environment:**  Leadership behaviours demonstrated by PE teachers (transformational teaching) | Physical Activity Questionnaire for Adolescents (PAQ-A); **within-class PA** and **leisure time PA** | | - Positive (indirect) effects on within-class PA and leisure-time PA. Relationship between transformational teaching behaviour and adolescent PA mediated by affective attitudes (interest and enjoyment). |
| 109 | Bryan et al (2012)  USA  O(CS)  * | 114 adolescents in grades 6-8 from 1 middle school | **Social environment:** Motivational climate; learning and performance orientation | Pedometer steps (3 PE days; **within-class PA,** and 1 24-hour count; **total PA**  Physical Activity Questionnaire for Children (PAQ-C); **total MVPA** | | - No association between motivational climate and PA (self-report or pedometer steps). |
| 110 | Cawley et al (2007)  USA  O(CS)  *** | 36,833 adolescents in grades 9-12. | **Policy environment:**  State-level PE regulations (including: PE binging unit requirement, PE exemptions, fitness testing policies etc.) | Self-reported number of minutes spent active in PE; **within-class PA** (Youth Risk Behavior Surveillance System; YRBSS) and number of days engaged in light, vigorous and strength building exercise (CDC healthy people); **total PA** | | - A binding PE unit is associated with greater participation in PE, but less activity in PE in boys. - PE exemptions for sports participation associated with more minutes of activity in PE. - A binding PE unit requirement is associated with more days of vigorous exercise and days of strength building in girls only - PE exemptions for sports participation associated with fewer days of light activity in boys and fewer days of strength building exercise in boys and girls. - PE testing associated with fewer days of strength building in boys and girls. |
| 28 | Cecchini et al (2014)  Spain  E  ** | 447 adolescents aged 12-17 (grades 9-12; mean age = 14.3 years) from 8 secondary schools | **Social environment:** Motivational climate TARGET strategies; task (design of activities); authority (decision making); recognition (use of rewards); grouping (group formation); evaluation (criteria) and time (pace of instruction) | Leisure Time Exercise Questionnaire (LTEQ); **leisure time total PA** | | - Significant increases in leisure time PA were observed for the experimental group only at post-1 (immediately post-intervention) and post-2 (3 months). |
| 29 | Chatzisarantis et al (2009)  England  E(cRCT)  *** | 215 adolescents aged 14-16 (grades 9-12; mean age 14.8 years) from 10 secondary schools | **Social environment:** Autonomy supportive environment  Positive feedback (based on effort not performance); providing rationale and choice and acknowledging difficulties | Leisure Time Exercise Questionnaire (LTEQ); **leisure time vigorous PA** | | - Positive effects for intervention group only on vigorous leisure time activity. |
| 111 | Chow et al (2009)  Hong Kong  O(CS)  ** | 123 PE classes from 30 secondary schools (grades 7-12) | **Physical and Policy environment:**  Class size, lesson location, size of instructional area. | Teacher rating of student PA (% being active and average intensity); **within-class PA** | | - No association of class size and student PA - No association between lesson location and student PA. - Positive association between activity area and student PA. |
| 30 | Gao et al (2011)  USA  O(P)  *** | 194 adolescents in grades 6-8 (mean age = 12.4 years) from 1 middle school | **Social environment:**  Motivational climate | Actical activity monitors measured **within-class MVPA** | | - Mastery climate associated with greater in-class activity. - Performance climate did not predict in-class activity. - Relationship between motivational climate and PA is mediated by self-efficacy. |
| 118 | Hagger et al (2003)  UK  O(P)  *** | 295 adolescents aged 13-16 (mean age = 14.5 years) from 3 secondary schools | **Social environment:**  Autonomy support in PE (teaching behaviours) | LTEQ; **vigorous leisure time activity** | | - Positive association between autonomy support in PE and leisure time vigorous PA - A path analysis revealed that perceived autonomy support in physical education affected leisure-time physical activity directly and indirectly through a motivational sequence involving internal perceived locus of causality, attitudes, perceived behavioral control, and intentions. |
| 72 | Hagger et al (2005)  UK, Greece, Poland and Singapore  O(P)  *** | 551 adolescents from 8 schools (UK, Greece, Poland and Singapore) | **Social environment:**  Autonomy support in PE (teaching behaviours) | LTEQ; **vigorous leisure time activity** | | - No direct path between autonomy support and PA. - A path analysis revealed a path from autonomy support in PE via autonomous motives (in leisure time) and TPB variables to leisure time PA. - PAS in PE had significant total effects on autonomous motives in LT, except in the Polish sample. |
| 121 | How et al (2013)  Australia  E  ** | 257 year 8 adolescents (mean age = 12.9 years) from 1 secondary school | **Social environment:**  Provision of choice (choice of preferred participatory role in PE unit: 1. usual role; 2. PE development officer (coach/umpire) or 3. plan own PA option) | **PA during PE class**; actigraph (MVPA and VPA) | | - Intervention group demonstrated greater MVPA in class (particularly those choosing their own PA plan) - Male students in intervention condition who chose option 3 were more active than controls and intervention option 1 and 2. - Female students in intervention condition who chose option 3 were more active than controls and intervention option 2 (no difference between option 3 and 1). |
| 73 | Jaakkola et al (2013)  Finland  O(P)  *** | 237 adolescents in grades 7-9 (13 years old in grade 7) from 8 secondary schools | **Social environment:**  Motivational climate | Self-reported engagement in **total PA** (MVPA and VPA) | | - No significant associations (correlation) between motivational climate in grade 7 and PA in grade 9. - A path analysis revealed a path from task-involving motivational climate via perceived competence and intrinsic motivation to self-reported physical activity. - Perceived competence and intrinsic motivation were significant mediators of task-involving climates and PA. |
| 74 | Jackson et al (2013)  Singapore  O(P)  *** | 990 adolescents in grades 7-9 (mean age 13.9 years) from 5 high schools | **Social environment:**  Relatedness support | LTEQ; **leisure time PA** (vigorous, moderate and mild PA) | | - Positive association for relatedness support on LTPA. - Students reported stronger other-efficacy (confidence in teachers abilities) and RISE beliefs (beliefs about teachers confidence in their (i.e., the students) abilities) when they felt that their teacher created a highly relatedness-supportive environment. - Student’s relational efficacy beliefs supported their confidence in their own ability, and (directly and indirectly) predicted more autonomous motives for participation in PE, and displayed prospective effects in relation to leisure-time PA. |
| 112 | Jin et al (2013)  South Korea  O(CS)  **** | 572 adolescents in grades 6-8 from 8 middle schools. | **Social environment:**  PE teacher behaviours (feedback, prompts, demonstrations, out of class encouragement, no PA promotion) | Pedometer recorded PA (steps); **within-class PA** | | - No association between PE teacher PA promotion behaviours and within-class PA. |
| 25 | Lonsdale et al (2013)  Australia  E(cRCT)  **** | 288 grade 8 adolescents (mean age 13.6 years) at 5 secondary schools | **Social environment:**  Teaching strategies: Relevance (explaining rationale and importance of activity); Providing choice (providing students with 2-4 opportunities for choice in the lesson); Free choice (providing the students with equipment, but refraining from giving instructions) | Actigraph measured **within-class PA** (proportion of time in MVPA and sedentary) | | - The ‘free choice’ intervention increased within class PA. - ‘Providing choice’ and ‘free choice’ decreased sedentary behaviour. |
| 36 | McKenzie et al (2000)  USA  O(CS)  **** | 126 PE teachers observed in 24 middle schools | **Physical and Policy environment:**  Class size and indoor versus outdoor lessons | System for Observing Fitness Instruction Time (SOFIT); **within-class PA** | | - Class size negatively associated with PA. - Class size positively associated with time spent sitting. - Although mean energy expenditure did not differ between indoor and outdoor lessons, students were less likely to be sitting during outdoor PE. - Students are more likely to be standing and walking in outdoor lessons, but the % being very active was higher in indoor lessons. |
| 37 | McKenzie et al(2004)  USA  O(CS)  **** | 298 PE lessons observed (26 boys only; 32 girls only and 240 co-ed) from 24 middle schools | **Policy environment:**  Single sex versus co-ed PE | System for Observing Fitness Instruction Time (SOFIT) **within-class PA** | | - Boys only PE classes and co-ed PE classes demonstrated more PA than girls only classes - Trend for girls to be more active in co-ed (but not statistically significant). - No difference between single sex and co-ed class activity for boys. |
| 120 | McKenzie et al (2004)  USA  E(RCT)  *** | 1849 PE lessons observed (214 PE teachers) from 24 middle schools | **Social environment:**  Instructional strategies to enhance PA in PE. | System for Observing Fitness Instruction Time (SOFIT); walking and vigorous intervals summed to indicate **within-class MVPA**. | | - The intervention increased student MVPA in PE. - Effects were cumulative; by year 2, intervention schools had further increased PA. - Effect sizes were greater for boys than girls. |
| 75 | Moreno-Murcia et al (2013)  Spain  O(CS)  ** | 698 adolescents aged 1216 (mean age = 14.2 years) from 10 secondary schools | **Social environment:**  Autonomy support | Habitual PA questionnaire; leisure time sport and leisure time activity (non-sport) subscales only; **leisure-time (total) PA** | | - Positive association (correlation) between autonomy support; praising autonomous behaviours and interests in athletes input and PA - Relationship between autonomy support and PA mediated by need satisfaction, intrinsic motivation and intention. |
| 113 | Parish et al (2003)  USA  O(CS)  ** | 442 adolescents in grades 6-8 (mean age = 12.6 years) from 1 middle school | **Social environment:**  Motivational climate | Pedometer (step count); **within-class PA** | | - After controlling for gender and ability, perceptions of a mastery climate associated with greater PA. |
| 122 | Perlman et al (2013)  Australia  E  ** | 84 adolescents in grades 11-12 (mean age = 16.3 years) from 1 secondary school | **Social environment:**  Autonomy supportive, controlling or balanced environment | Actigraph (**within- class PA**; MVPA and total PA) | | - Significant differences for students in the autonomy supportive context in terms of MVPA - No difference between controlling and balanced environments. |
| 119 | Pihu et al (2008)  Estonia  O(P)  *** | 399 adolescents (mean age = 14.7 years) from 5 secondary schools | **Social environment:**  Positive general feedback | LTEQ; **vigorous leisure time PA** | | - Positive association between teachers’ positive general feedback and leisure time PA. |
| 123 | Schuldheisz et al (2001)  USA  E(ssRD)  * | 8 “low activity” adolescents in grade 7 from 1 middle school | **Social environment:**  Active supervision: frequent interactions with students and higher rates of movement  Passive supervision: no verbal prompts, no encouragement, little movement | System for Observing Fitness Instruction Time (SOFIT); **within-class MVPA** | | - Mean MVPA levels were higher during the active supervision (not statistically tested). |
| 77 | Slingerland et al (2013)  The Netherlands  E  ** | 216 adolescents in grades 7-9 (11-15 years) from 13 classes in 1 secondary school | **Policy environment:**  single-gender versus co-ed game play in a PE lesson | Polar Team System heart rate monitors; **within-class MVPA** | | - Physical activity levels did not change during single-gender game play (compared to co-0ed game play) - Playing in single gender teams increased girls’ perceived competence levels and decreased their perception of competitiveness. |
| 76 | Zhang et al (2011)  USA  O(CS)  * | 286 adolescents in grades 6-8 (mean age = 13.4 years) from 1 middle school | **Social environment:**  Psychological need support (autonomy, competence and relatedness support) | Physical activity questionnaire for older children (PAQ-C); **total MVPA** | | - Positive correlation between need support in PE and PA. - Need satisfaction and intrinsic motivation mediated the relationship between need support and physical activity. |
| 114 | Zhang et al (2012)  USA  O(CS)  ** | 285 adolescents in grades 6-8 (mean age 13.4 years) from 1 middle school | **Social environment:**  Social support from PE teachers (fair treatment, active participation, encouragement/praise, giving individualized help and listening to needs and concerns) | Physical activity questionnaire for older children (PAQ-C); **total MVPA** | | - Positive correlation between PE teacher support and PA. - Teacher support significant predictor of PA after controlling for other variables. |

Quality assessment (* ratings) details provided in Table S2. ^ᴛ^ = only cross-sectional analysis findings reported in this review as longitudinal analyses include primary school aged children. E = experimental design, O = observational design, CS = cross-sectional design, P = prospective design, RCT = randomised controlled trial cRCT = cluster randomised controlled trial, ssRD = single-subject reversal design * Longitudinal data also reported but given that this includes primary school aged children, we exclude this data.

**Table S4. Overview of qualitative studies**

|  | Study ID | Aim of study | Sample and Methods | Key findings – factors influencing PA in schools |
| --- | --- | --- | --- | --- |
|  | *Whole school environment* | | | |
| 54 | Bauer et al (2004)  USA  *** | To identify factors in school physical and social environments that may facilitate or compete with programs and policies to improve student PA (and nutrition) | 26 students (grades 7-8) and 23 faculty from 2 middle schools  Focus groups (students and teachers); interviews with key informants (e.g., administrators, school nurses) | **Competition** (competitive PE environment) reduces participation, especially for female and/or low athletic ability  **Open gym policies** (supervised but unstructured activity) has positive effects on participation (but mostly for boys)  **Extracurricular sport** is exclusive due to limited coaches/space/equipment, therefore limited to athletically competent students; few opportunities to learn sports skills (so those making teams are already skilled/regularly active)  **Active travel policies** discourage unsupervised active transport (due to stranger danger) and no crossing guards also discourage active travel) |
| 56 | Booth et al (2008)  Australia  ** | To explore how adolescents perceive overweight and obesity, it’s causes and solutions: extent to which young people feel the secondary schools should play an active role in prevention and the key changes/support required to reduce overweight and obesity, | 58 adolescents aged 12-17 from four secondary schools (3 urban and 1 rural school)  Focus groups with students | **Competition** focussed environment overrides focus on participation and this attitude puts students off participating. Students emphasised encouragement and participation rather than competition and exclusion for school PA.  **Academic pressures** – the focus on academic work meant fewer opportunities for activity/sport.  Students believed schools should increase amount of structured PE and time spent being active in PE (rather than taking notes)  **Rules and regulations** – students thought there should be more consequences for those trying to avoid PE  **Use of space** could be improved (i.e., improving existing sports areas) and **access to sports equipment** during lunch and recess is important for PA participation. |
| 45 | Boyle et al (2008)  UK (England)  *** | To gain an understanding of adolescents PA levels from the perspective of those responsible for delivering PE in schools. | 17 heads of PE and two heads of staff (secondary schools)  Semi-structured interviews | **Time constraints for delivering PA in the curriculum** - very few schools had policies whereby all pupils received 2 hours of core PE per week; this got worse as students got older.  **Health-related exercise confusion**- attitudes that exercise equals competition – unclear of whether this fitted into PE or separate block.  **Ethos of performance/elitism** within PE department or school as a whole: Competing interests in PE department- desire to raise profile of school through sporting excellence versus encouraging mass participation.  **Undervaluing activities -** Lack of volunteering by other school staff for extracurricular sport (e.g., supervision at lunch times); perception that senior management prioritised PE than other academic subjects.  **Encouragement of PA outside curriculum:** **facilities** within school and community considered important for PA participation. |
| 46 | Dagkas et al (2007)  UK (England)  *** | To explore the social factors that influence young people’s participation in school and out of school activities | 52 adolescents from two diverse secondary schools (1 suburban school ‘A’ and one inner city school ‘B’)  Group interviews | **Access to provision, opportunity and location:** School A promoted involvement inactivity outside of school by encouraging students to join clubs and establishing links with local clubs  **Lack of physical infrastructure:**  School B had limited facilities meaning the school could offer very few activities  **Encouragement and motivation:** PE teacher support and encouragement is important (i.e., explaining benefits of healthy life) |
| 57 | Dwyer et al (2006)  Canada  ** | To explore perceived barriers to participation in moderate and vigorous PA among adolescent girls who live in a large ethno-racially and socioeconomically diverse city | 73 female adolescents aged 15-16 from four secondary schools  Focus groups | **Teacher discouragement** for girls participating in sports (especially sports such as football)  **Lack of opportunity to take PE classes** especially when schools only offer PE for 1 semester/PE not compulsory after grade 9/PE not an option in an academic stream  **Outdated equipment**  **Competitive element to school sport** discourages participation for most students (for some it encourages participation) |
| 38 | Goh et al (2009)  USA  **** | To explore adolescent, parent and community stakeholder perspectives on barriers to healthy eating and PA and intervention ideas to address obesity | 119 adolescents in grades 7-8 from two middle schools, 63 parents and 28 stakeholders  Focus groups (students and parents); interviews with stakeholders | **Inadequate quantity and/or quality of PE** e.g., large class sizes  **Non-competitive activities** important for PA  **Access to better facilities and equipment** during recess/lunch/after-school |
| 47 | Hannay et al (2013)  USA  *** | To engage Latina teens and their parents in identifying barriers to PA and initiating policy changes to address them | 36 female adolescents aged 14-19 and 41 adults (parents of adolescents) from two secondary schools.  Focus groups and photovoice | **PE policies** – PE only required in grade 9 in some schools (not for whole school)  Non-participation in PE related to not wanting to participate with boys, not wanting to change early in the day and disgust at poor condition of locker rooms.  Separating PE classes by gender or scheduling PE at the end of the day would facilitate participation  **Non-competitive activities** wanted by girls. |
| 58 | Hohepa et al (2006)  New Zealand  *** | To explore the views high school students have about various PA contexts and their ideas about potential PA promoting strategies | 44 adolescents aged 13-15 from 3 secondary schools  Focus groups | **Accessibility and availability** – the lack of equipment to borrow/nothing to do at lunchtimes  **Structure of PA opportunities**: Focus on sports and lack of social teams limits participation for most adolescents/only ‘good’ people take part; require more organised activities at lunchtime |
| 53 | Hyndman et al (2012)  Australia  *** | To explore the broader influences on students’ PA behaviour beyond the classroom | 22 adolescents for focus groups and 7 students for map drawings* from two secondary schools  Focus groups and map drawings | **Built environment** – students ‘too old’ for playgrounds, perceptions that ‘safe; play spaces are boring; territorial issues in the playground limit PA, spacious environments promote PA.  **Supervision** – too much supervision negatively impacts activity  **Lack of access to sports equipment** (e.g., difficulty borrowing equipment)  **Policies** limit PA, such as no access to play/sports facilities (unsupervised) |
| 48 | Kirby et al (2009)  UK (Scotland)  ** | To explore the views of school children on active travel to school and their ideas about promotion strategies for school-based interventions | 41 adolescents aged 10-13 from 3 secondary schools  Focus groups | **Bike safety at school** – fears of vandalism and theft/bikes not safe if taken to school  Requirement for bike storage **facilities** and **cycle proficiency training/reward and incentive** policies for students to promote active travel.  **Teacher role modelling** for active travel is important |
| 49 | Kirby et al (2013)  UK (Scotland)  ** | To identify environmental factors that influence PA participation among young people | 131 adolescents for computer session and 63 for focus groups; aged 11-13 from six secondary schools | **Lack of facilities** meant that students have to commute offsite for PA which limits activity time, also commuting by bus/coach to off-site PA (instead of walking)  **Poor changing facilities**  **Facilities that can be used out of hours** – especially in areas with limited public facilities would promote PA  **PE provision and structure** – students reported a need for greater PE time; mixed options about gender segregated versus co-ed PE  **School design** – One school had a lot of stairs so walking around the school was described as the PA routine. |
| 63 | Knowles et al (2011)  UK (Scotland)  *** | To explore factors related to the decrease in physical activity behavior in adolescent girls during the transition between primary and secondary school. | 14 females adolescents (mean age = 13.6 years) from 6 secondary schools  Semi-structured interviews | **Unsupportive social environment** for PA in secondary school, such as feeling uncomfortable in a competent PA environment and the creation of a more competitive environment at secondary school.  **School culture –** At secondary school, being active at break and lunch times was not part of their school culture, which created an unsupportive PA environment. |
| 55 | Kubik et al (2005)  USA  *** | To increase the understanding of factors that may influence the (dietary and) physical activity practices of adolescents attending an alternative high school. | 70 adolescents in 9^th^-12^th^ grades from alternative high schools  Focus groups | **Social support** and **role modelling** of friends and adults (i.e., teachers) helped students to be active.  **School scheduling** left many students too tired and too busy for activity.  **Limited space and equipment** was also identified as reasons for not being active.  To promote active students wanted **more opportunities to be active** in the school day and **more freedom to choose** activities, a desire for **more outside time** and **access to exercise facilities after school.** |
| 39 | MacQuarrie et al (2008)  Canada  **** | To provide an interpretation of how adolescents, parents, teachers and principals perceive students’ involvement in physical activity within their intermediate school environment. | 6 students, 6 teachers, 3 principals and 3 parents from 3 middle schools.  Semi-structured interviews | **School culture –** Students, teachers, principals and parents recognized that how a school approached the notion of PA had an important impact on how active the people in the school were. The extent to which a school involves everyone from students through to administration in the planning and carrying out of ways to engage everyone in active lifestyles, the stronger the school culture on the promotion of physical activity.  **Teacher encouragement** and **modelling** of activities was felt to be important for participation.  **Building opportunities for physical activity into the curriculum and making physical activity a priority** were seen as instrumental in crafting a culture of physical fitness but competing time demands make this difficult.  **Intramurals** and **monitored activities** are an important part of schools culture as they signify the value of physical activity. Intramurals create an atmosphere of playfulness, and encourage those who don’t want to take part in organised sports.  A **focus on elitism (placing higher value on athletic elitism)** may be detrimental to PA engagement; athletic elitism can fracture the student population into subgroups whose sense of belonging will vary depending on how much they feel they are important and connected to the school. Students’ **judgments of belonging** are core to motivating students involvement in physical activity.  **Gender segregation** for physical activities was generally supported by the teachers, especially for girls participation |
| 50 | Monge-Rojas et al (2009)  Costa-Rica  ** | To assess the perceptions of rural and urban Costa-Rican adolescents regarding which barriers and motivations affect their adoption of an active lifestyle | 108 adolescents in grades 7-11 from 3 secondary schools  Focus groups | Participants perceived that the **PE environment** was uninteresting and placed too much emphasis on competitive team sports  The school environment lacked suitable **areas and facilities** to practice physical activity.  **Facilities are not open after school hours** which limit participation.  Students thought that teachers (and parents) need to become better **role models** to facilitate a stronger **social support** network for activity. |
| 78 | Moore et al (2010)  USA  *** | To elicit socio-ecological barriers and facilitators for PA in rural and urban middle school youth and their parents. | 41 6^th^ grade youth (mean age = 12.6 years) and 50 parents from 3 middle schools.  Focus groups | **School physical activity policies** mentioned as a barrier to activity: PE scheduled for only half of the semester (‘health’ was covered in the other half); age requirements existed for participation in school sports which also limits opportunities for involvement; policies on which grades get to participate in outdoor recess limits time for activity.  Students reported that the amount of homework assignments limits time spent in physical activities; teachers make excuses to skip recess (e.g., if work is not completed in class). |
| 59 | Robbins et al (2009)  USA  *** | To explore perceived benefits, barriers, self-efficacy, enjoyment or activities preferences, and situational or environmental influences related to physical activity. | 40 male adolescents aged 11-13 from 2 middle schools.  Focus groups | **Inadequate number and poor condition of sports equipment**.  Participants felt that the school environment lacked opportunities for activity, particularly for boys. Boys perceived that more programs were being created for girls and that activities that boys liked (e.g., martial arts/ice hockey) were too costly.  **The absence of recess** and **limited opportunities to take PE or gym were cited as barriers.** Students often opted for arts or music above PE or gym class.  Boys emphasised the need to make physical activity more fun; increasing the competitive element (making into a game or contest), setting up obstacle courses, and using **rewards** for reaching activity goals and being active.  Boys emphasised that they wanted ‘freedom’ to do activities and to not feel forced into activities, such as running.  Boys also emphasised the need for wider **social support and encouragement** from adults in the school, such as the school Nurse (e.g., to receive more support and encouragement for PA). |
| 64 | Slater et al (2010)  Australia  *** | To gain a deeper understanding of the reasons that adolescent girls give for ceasing participation in sport and other physical activities and why they do not participate as much as boys. | 49 female adolescents aged 13-15 from 2 secondary schools.  Focus groups | **The range of sporting options available to girls** were significantly less than those available to boys (e.g., boys have more sports to choose from and more teams and divisions to cater for a range of abilities.  **Uniforms** for girls activity cause concerns about body image and can lead to less participation in activities.  **Competitive environment** for sports at secondary school makes activities less enjoyable.  Perceptions that playing sports is immature for girls in secondary schools. |
|  | *PE environment only* | | | |
| 40 | Azzarito et al (2006)  USA  **** | To examine ways in which high school girls participated in or restricted PE. | 15 female adolescents (9^th^-10^th^ grade) from 3 classes in 1 secondary school and 1 PE teacher  Interviews with students and teacher, informal chats with teachers and field notes | **Co-ed PE**- Girls were positive about co-ed PE and felt able to improve themselves when interacting with boys. This is dependent on the activity girls are participating in – crucial to have a learning environment where girls feel supported in their participation and comfortable with their skill level.  **Choice in PE**- Girls restricted their engagement with PE when they *perceived it as being* gendered, unwelcoming to their participation and thus, not a choice. |
| 67 | Constantinou et al (2009)  USA  *** | To determine how middle school girls perceive their PE teachers' gender-role expectations and how these perceptions affect the girls' participation in and attitudes toward physical education. | 20 female adolescents (of varying skill level) in 7^th^ - 8^th^ grade from 1 middle school.  Interviews with adolescents and informal interviews with teachers and field notes. | **Grading policies in PE**- Grading systems that focus on effort rather than skills increases effort and participation in PE  **Co-ed PE perceptions-** Girls wanted boys on teams as they believed in made them work harder. However girls reported negative feelings about participation when boys created physically or emotionally unsafe learning environments.  **Competitive atmosphere in PE-** Girls attributed their participation and attitude towards PE to a ‘competitive atmosphere’ which was considered a positive feature of PE (however, some girls noted that it was a problem, however, if boys became overly competitive). |
| 51 | Hassandra et al (2003)  Greece  *** | To provide further information regarding factors associated with students intrinsic motivation in PE | 16 adolescents aged 12-15 from 2 secondary schools  Semi-structured interviews | **PE teacher teaching behaviours** affects motivation and participation: students with differing levels of intrinsic motivation appeared to have very different perceptions of PE teacher supportiveness within lessons  **School athletic facilities** – students commented that the lack playgrounds and athletic facilities limited play |
| 41 | Lamb et al (2014)  UK  **** | To explore the impact of ‘excuse notes’ focusing on participation levels within PE lesson. | Parents, PE teachers (n=12), trainee PE teachers (n=37) and students (n=58) from 10 secondary schools adopting a policy of excuse notes.  Surveys, focus groups, fields notes, brief case studies and analysis of excuse notes. | Excuse note policy may be a barrier to learning and participation in PE.  **Excuse note policy –** Writing an excuse note is deemed an acceptable policy which provides a mechanism for pupils to self-exempt from PE. This ritual reinforces the value that PE does not hold much value or priority.  As the excuse note holds a place within the infrastructure of the PE department policy, it does not warrant challenging in the same way that simply ‘refusing to take part’ would.  Students acknowledge that the excuse note provides a means for them to disengage from PE.  A policy in which pupils would still be asked to change for PE, irrespective of an excuse note, was supported by 50% of parents. |
| 65 | Morton et al (2010)  Canada  *** | To examine the extent to which transformational leadership behaviours were displayed by PE teachers and to explore how perceptions of transformational teaching were aligned to students cognitive, affective and behavioural responses in PE. | 62 adolescents aged 13-15 from 3 secondary schools.  Focus groups and follow up semi-structured interviews | Students who perceived their teacher to utilise **transformational leadership** behaviour described more adaptive responses, both within class activity and out of school activity.  **PE teacher encouragement and enthusiasm** (‘inspirational motivation’) associated with activity in class and outside of class.  **PE teacher leadership and role-modelling behaviours** (‘idealized influence’) associated with activity behaviour in class. PE teacher respect discussed in relation to activity outside of school.  **PE teacher supportiveness and caring behaviours** (‘individualized consideration’) associated with skill development improvement and involvement in PE classes.  **Providing rationale** for activities, rather than forcing activity in PE associated with ore adaptive responses in PE. |
| 52 | Ntounamis et al (2004)  UK  *** | To provide an in depth account of amotivation in compulsory school PE by examining its causes, ways in which its displayed and how it can be tackled. | 21 adolescents aged 14-15 years from 4 secondary schools. All students were identified as ‘amotivated’’ for PE.  Semi-structured interviews | **PE policy – time allocated to PE** meant that it cannot achieve its purpose; there is a need for PE to have more of a central role in the school’s curriculum.  Physical environmental factors, such as **poor weather conditions** and **poor facilities,** undermine motivation for participation.  **Teaching style** – inappropriate teaching attitudes and behaviours contributed to poor student/teacher relationships. **Exercise as punishment** does not achieve the desired effect of more effort; it induces feelings of anger and injustice and lowers motivation.  **Motivational climate** – students perceived a climate that had little emphasis on learning and improvement; attention is given to the most competent students and praise was only provided when students outplayed their peers.  Students would like teachers to make them feel more involved and give students more choice; teacher should provide more **praise** in PE  Students expressed a **desire for groups of similar ability** so that intragroup differences would be less salient.  Some girls proposed that students should have a choice of gym wear and be given more time to change. |
| 66 | Smith et al (2009)  USA and UK  *** | To identify American and English secondary school determinants of enjoyment in PE. | 15 adolescents aged 14-15 from 1 American secondary school and 15 adolescents from 1 UK secondary school. All students had high enjoyment of PE.  Semi-structured interviews | **Teacher attitudes and personalities** – Students believed that enjoyment of PE was enhanced when teachers possessed enthusiasm (passion and excitement) for teaching the subject; a caring persona also influenced enjoyment of PE, as did having a sense of humour.  Students also reported liking when the teacher joins in with activities (**role modeling**).  **Verbal interaction** – the teachers ability to communicate effectively (e.g., provide encouragement and positive feedback) was important to students.  **Competition** – male students saw competition as a positive and motivating factor for participation. Although females commented on positives of competition they also discussed negative aspects, such as removing the ‘fun’ from activity.  Students had mixed views about splitting the classes by ability. |

Quality assessment (* ratings) details provided in Table S2. * we excluded the primary/elementary school student data
